# Molecular mechanisms of the non-coenzyme action of thiamin in brain: biochemical, structural and pathway analysis

**DOI:** 10.1038/srep12583

**Published:** 2015-07-27

**Authors:** Garik Mkrtchyan, Vasily Aleshin, Yulia Parkhomenko, Thilo Kaehne, Martino Luigi Di Salvo, Alessia Parroni, Roberto Contestabile, Andrey Vovk, Lucien Bettendorff, Victoria Bunik

**Affiliations:** 1 Faculty of Bioengineering and Bioinformatics of Lomonosov Moscow State University, Leninskije Gory 1, 119992 Moscow, Russian Federation; 2 Department of Vitamin and Coenzyme Biochemistry, Palladin Institute of Biochemistry, NAS of Ukraine, 9 Leontovicha Street, 01601 Kyiv, Ukraine; 3 Institute of Experimental Internal Medicine, Otto-von-Guericke University Magdeburg, Germany; 4 Dipartimento di Scienze Biochimiche “A. Rossi Fanelli”, Sapienza Università di Roma, via degli Apuli 9, 00185 Roma, Italy; 5 Institute of Bioorganic Chemistry and Petrochemistry National Academy of Sciences of Ukraine, Kyiv, Ukraine; 6 GIGA Neurosciences, University of Liege, Quartier Hôpital, Avenue Hippocrate 15, 4000 Liege, Belgium; 7 Belozersky Institute of Physicochemical Biology of Lomonosov Moscow State University, Leninskije Gory 1, 119992 Moscow, Russian Federation

## Abstract

Thiamin (vitamin B1) is a pharmacological agent boosting central metabolism through the action of the coenzyme thiamin diphosphate (ThDP). However, positive effects, including improved cognition, of high thiamin doses in neurodegeneration may be observed without increased ThDP or ThDP-dependent enzymes in brain. Here, we determine protein partners and metabolic pathways where thiamin acts beyond its coenzyme role. Malate dehydrogenase, glutamate dehydrogenase and pyridoxal kinase were identified as abundant proteins binding to thiamin- or thiazolium-modified sorbents. Kinetic studies, supported by structural analysis, revealed allosteric regulation of these proteins by thiamin and/or its derivatives. Thiamin triphosphate and adenylated thiamin triphosphate activate glutamate dehydrogenase. Thiamin and ThDP regulate malate dehydrogenase isoforms and pyridoxal kinase. Thiamin regulation of enzymes related to malate-aspartate shuttle may impact on malate/citrate exchange, responsible for exporting acetyl residues from mitochondria. Indeed, bioinformatic analyses found an association between thiamin- and thiazolium-binding proteins and the term *acetylation*. Our interdisciplinary study shows that thiamin is not only a coenzyme for acetyl-CoA production, but also an allosteric regulator of acetyl-CoA metabolism including regulatory acetylation of proteins and acetylcholine biosynthesis. Moreover, thiamin action in neurodegeneration may also involve neurodegeneration-related 14-3-3, DJ-1 and β-amyloid precursor proteins identified among the thiamin- and/or thiazolium-binding proteins.

The knowledge of the molecular mechanisms underlying the pharmacological effects of drugs is indispensable to improve their safety and efficiency. The identification of molecular targets of pharmacologically active compounds is an important step to understand such molecular mechanisms, and is greatly advanced by modern development of high-throughput analytical and bioinformatics approaches. Thiamin (also known as vitamin B1) is widely used in neuropharmacology. In particular, its administration causes a transient improvement in cognitive function of some patients affected by neurodegenerative diseases, including Alzheimer’s disease (AD) and Parkinson’s disease (PD)^1^,^2^,^3^,^4^,^5^. The importance of thiamin administration in elderly is supported by the fact that the levels of thiamin and its coenzyme form, thiamin diphosphate (ThDP), are decreased with age^6^. In patients with neurodegenerative diseases, such as AD and fronto-temporal dementia, significantly less ThDP than in the age-matched control group was determined in post-mortem cortex samples^7^,^8^. Several features of thiamin pharmacology are worth noting. First, rather high doses of this vitamin (e.g. app. 14- and 90-fold excesses over the recommended daily dose in a Vitamin B-Komplex of Ratiopharm GmbH, Germany, and Neuromultivit of Lannacher Heilmittel GmbH, Austria, respectively) can be employed in medical practice, as they are not known to have adverse effects. Second, apart from the widely accepted ThDP action as a coenzyme of central metabolism, thiamin has long been known to co-release with acetylcholine^9^,^10^,^11^ facilitating synaptic transmission^12^. Independent studies suggested the involvement of proteins of synaptosomal plasmatic membrane hydrolyzing the non-coenzyme derivative thiamin triphosphate (ThTP)^13^,^14^,^15^,^16^,^17^ and phosphorylating synaptic proteins with ThTP as a phosphate donor^18^. Yet, the identification of either molecular targets of this non-coenzyme action of thiamin, or proteins metabolizing the non-coenzyme derivatives of thiamin, is far from completion. A new cell membrane ThDP transporter^19^ and poly(ADP-ribose) polymerase-1 (PARP-1) regulated by adenylated ThTP^20^, have recently been added to the five known mammalian proteins of thiamin metabolism, such as the two thiamin transporters of cell membrane, ThDP transporter of mitochondria, soluble thiamin triphosphatase and thiamin diphosphokinase. However, the enzymes producing adenylated thiamin di- or triphosphates^21^ and the other mammalian targets of thiamin non-coenzyme forms were neither purified to homogeneity, nor identified at molecular level^12^. This greatly hinders the understanding of the molecular mechanisms of non-coenzyme action of thiamin and its derivatives *in vivo*. Also ThDP has non-coenzyme functions beyond its coenzyme role. For instance, it affects protein translation, either as riboswitch in thiamin-synthesizing species (plants and bacteria), or through regulation of p53 binding to DNA in mammals^22^. Insofar, natural thiamin derivatives may be pharmacologically significant not only due to the well-known coenzyme role of ThDP in central metabolism. Another important aspect is that pharmacological compounds often possess heterocycles which are structurally similar to those present in thiamin and derivatives, and may therefore act by targeting thiamin-dependent pathways^23^. In particular, drugs which reduce hyperphosphorylated tau-protein in AD mouse models^24^ possess structural similarity to thiamin and may therefore mimic or interfere with the pathways of the thiamin non-coenzyme action in synaptic transmission. The existence of such pathways, in addition to the known metabolic role of ThDP, could explain the absence of a robust correlation between positive effects of thiamin in patients with neurodegenerative diseases and activities of ThDP-dependent enzymes and ThDP levels in the brain of these patients^13^,^25^. Our work aims at the molecular identification of proteins and pathways involved in the non-coenzyme action of thiamin compounds. To do so, we used a previously established protocol to obtain a fraction of brain synaptosomes enriched with thiamin binding and thiamin phosphates hydrolyzing proteins^14^,^15^,^17^,^26^. The fraction was subjected to affinity chromatography on thiamin- or 3-decyloxycarbonylmethyl-4-methyl-5-(2-hydroxyethyl) thiazolium (DMHT)-modified sorbents. The latter sorbent includes a decyloxycarbonylmethyl moiety attached to 4-methyl-5-(2-hydroxyethyl)-thiazolium heterocycle. This could mimic membrane-directed hydrophobic interactions of the aminopyrimidine ring of thiamin, whereas the heterocycle is specific to thiamin, i.e. not known to occur in natural compounds other than thiamin in animals. In addition, the heterocycle of DMHT is structurally similar to the thiamin degradation product 4-methyl-5-(2-hydroxyethyl)-thiazole, which has been identified after thiamin injection in different mammalian tissues, including brain^27^. Because thiamin degradation was detected also in germ-free rats^28^, the process is obviously performed not only by intestinal microflora, but also by mammalian thiamin-degrading enzymes. The non-coenzyme role of thiamin, especially at high doses, may also depend on the role of thiamin degradation products. In good accordance with this suggestion, DMHT was earlier shown to affect neuromuscular junctions, apparently through its effects on associated currents of calcium and potassium ions^29^. The observation of the neurophysiological effects of DHMT *in vivo*^30^,^31^, however, was never extended to the identification of DMHT-binding proteins. In the present work, the enriched synaptosomal fractions collected upon elution from affinity chromatography were identified by tandem liquid chromatography–mass spectrometry (LC-MS/MS). The sets of proteins identified in the eluates from the thiamin- and DMHT-derivatized sorbents will hereinafter be mentioned as thiamin and thiazolium proteome, respectively. These partial synaptosomal proteomes were also analyzed by bioinformatics approaches, taking into account published data. Along with identification of known thiamin-dependent proteins and their heterologous protein partners, the analyses revealed a significant similarity between the protein components of thiamin and thiazolium proteomes. On the other hand, the common features of the two proteomes were shown to differ from those characteristic of other partial brain proteomes, such as the one containing DJ-1-binding proteins^32^ and the proteome of frontal cortex^33^. Non-random and common co-occurrences of the eluted proteins and protein clusters in thiamin and thiazolium proteomes favors specific interactions, both direct and protein-mediated, of our preparation of synaptosomal proteins with the thiamin and DMHT baits of the affinity sorbents used in this work. For the most abundant enzymes of both proteomes, the specificity was confirmed by kinetic studies, showing the effect of thiamin and its derivatives on the activity of these enzymes. Available structural data, together with kinetic analysis, were used to assess potential thiamin-binding sites in the enzyme 3D structures. As a result, targets of the thiamin non-coenzyme action in brain were shown to include the enzymes involved in metabolic communication between cytoplasm and mitochondria through malate-aspartate shuttle. Our bioinformatics analyses of thiamin and thiazolium proteomes also identified other thiamin-dependent pathways, including signaling through 14-3-3 proteins and calcium ions, redox defense and signaling involving peroxiredoxins and DJ-1 (parkin-7). Moreover, in view of the thiamin action in neurodegenerative diseases, the identification in the thiazolium proteome of β-amyloid precursor proteins is of particular interest.

## Materials and Methods

### Materials

Biochemicals, substrates and co-factors were from Sigma-Aldrich (Taufkirchen, Germany) and of the highest quality available. Commercial preparation of the purified glutamate dehydrogenase (GDH) from bovine liver (≥35 units/mg protein) and malate dehydrogenase (MDH) from porcine heart mitochondria (600–1000 units/mg protein) were from Sigma-Aldrich (Taufkirchen, Germany). Mass-spectrometry chemicals were: acetonitrile, LC-MS grade from VWR/Prolabo (Dresden, Germany); formic acid, ammonia carbonate and trifluoricacetic acid were from Fluka (Germany); Trypsin-Gold from Promega (Germany); methanol, glacial acetic acid and methoxyamine hydrochloride from Sigma-Aldrich (Taufkirchen, Germany); pyridine from Merck (Darmstadt, Germany). Cell culture media were from Gibco (Carlsbad, CA, USA). 3-decyloxycarbonylmethyl-4-methyl-5-(2-hydroxyethyl) thiazolium (DMHT) was obtained according to^30^. Thiamin triphosphate and its adenylated form were obtained as in^21^,^34^.

### Ethics Statement

The animal experiments conducted in the present study conform to the Guide for the Care and Use of Laboratory Animals published by the US National Institutes of Health (NIH Publication No. 85-23, revised 1996) and the EU Directives 86/609/EEC and 2010/63/EU. All animal experiments were approved by the institutional committees for animal care and use, such as Bioethics Committee of Moscow Lomonosov State University, Committee for animal care and use of the University of Liege and Committee for procuration, husbandry and usage of experimental animals of Palladin Institute of Biochemistry of NAS of Ukraine.

### Isolation of delipidated brain synaptosomal proteins

All procedures were done at 4–8 °C. Animals were kept at 21 ± 2 °C on standard ration without additional vitamin supplementation and 12/12 h light/dark cycle. 18–20 Wistar male rats of 200–250 g were killed by decapitation. Extracted brains (app. 1.5 g each) were cleaned from blood vessels on ice and homogenized in the ice-cold isolation buffer, i.e. 5 mM Tris-HCl buffer (tris(hydroxymethyl)aminomethane), pH 7.4, containing 0.32 M sucrose and 0.5 mM PMSF (phenylmethanesulfonylfluoride), using a Potter homogenizer with teflon pestle. Cell debris and nuclei were removed by a 10 min centrifugation at 1000 g. Crude synaptosomes including mitochondria were pelleted by centrifugation of the supernatant for 20 min at 15000 g. The pellet was suspended in 10 mL of the isolation buffer, followed by addition of 9 volumes of cold (−20 °С) acetone. The mixture was homogenized in the Potter homogenizer, and the suspension was incubated under moderate shaking for 10 min at 4 °С. The delipidated proteins were filtered on Buchner funnel, and washed on the glass filter with a double volume of ether. The protein pellet (so called “acetone powder”) was further dried at room temperature for 10–15 min and stored (up to several days) at −20 °С until affinity chromatography. To solubilize the proteins for chromatography, the acetone powder was mixed with 10 mM Tris-HCl buffer, pH 7.4, at a ratio of 20 mL of buffer per 1 g of powder. The mixture was homogenized in the Potter homogenizer and incubated under shaking for 30 min at 4 °С. Insoluble material was removed by a 10 min centrifugation at 20000 g. The solubilized proteins were diluted two-fold with an equal volume of the Ringer-bicarbonate buffer, pH 7.4 (final concentrations of the components in mM, are: NaCl 118, KH_2_PO_4_ 2.34, KCl 4.6, MgSO_4_•7H_2_O 1.19, CaCl_2_ 2.42, NaHCO_3_ 24.9, glucose 10), and the solution was subjected to affinity chromatography.

### Affinity sorbents

Thiamin- and thiazolium-binding proteins of brain synaptosomes were purified by affinity chromatography on sorbents carrying chemically bound thiamin or DMHT as bait. The baits were connected to diazotized sorbent-attached linker (7-(2,4-diaminophenyl)heptanoic acid hydrazide) through C2 atom of the thiazolium ring. The baits and the linkers were incorporated into the CNBr-activated Sepharose according to the described procedure^35^. Briefly, coupling of the linker to CNBr-activated Sepharose 4В was carried out in acetic acid for 8 hours at 4 °С. The resulting sorbent with the attached linker was washed with 50% dioxane and water, followed by diazotation through a 7 min incubation with 0.1 M sodium nitrite upon shacking at 4 °С. The diazotized sorbent was suspended in sodium borate buffer containing thiamin or DMHT, and рН of the suspension was adjusted to 8.6. Coupling of the baits to sorbent was performed for 4 hours at 4 °С, resulting in red products. The sorbents carrying the baits were washed with cold water and stored in aqueous suspension with 0.02% sodium azide. The bifunctional linker was used to enlarge conformational spectrum of the incorporated baits. Conformations of the baits in the two monosubstituted products could be different due to non-equivalence of their proximity to the hydrophobic linker. Modification of both diazonium groups of the diazotized linker was not supposed to occur to significant extent due to steric hindrance and relatively short (4 hours) modification time. This assumption agreed with no principal difference in the purification of the thiamin phosphate hydrolase activity using the sorbents whose linkers carried either one or two diazonium groups. The modification was terminated by washing out the reactants from the sorbent. The diazonium group which would not react with the bait within the modification time was deactivated by hydrolysis to the corresponding phenol.

### Affinity chromatography

The procedure was done according to an earlier protocol, which was elaborated to collect the fraction of synaptosomal proteins binding S^35^-thiamin and hydrolyzing thiamin phosphates, such as ThTP, ThDP and ThMP^26^. Solubilized proteins of the acetone-delipidated fraction of the crude rat brain synaptosomes were applied to a column with affinity sorbent (1.2 × 7 cm) equilibrated with Ringer-bicarbonate buffer, pH 7.4, at a rate of 2–3 mL per hour. Non-bound proteins were washed out until absorbance of the eluate at 280 nm approached background level. Bound proteins were eluted step-wise, at first with a 10 mM Tris-HCl buffer, pH 7.4, then with 1 M NaCl and finally with the same buffer containing 2 M urea, at a rate of 18 mL per hour. Hydrolysis of commercially available ThDP (ThDPase) was used to follow the elution of thiamin binding/hydrolase activity towards thiamin phosphates from the affinity sorbents. The fractions with the ThDPase activity were pooled, subject to rapid desalting on Sephadex G25, and dialyzed overnight against 10 L of 10 mM Tris-HCl buffer, pH 7.4. After dialysis, the proteins were lyophilized and stored frozen at −80 °С up to several months. During the period of biochemical analyses (a week), dissolved aliquots of lyophilized protein for experimental work were stored frozen at −20 °С. Affinity sorbents were regenerated by a step-wise washing with 8M urea and distilled water, and stored in 0.02% sodium azide.

### Proteome identification

Proteins in the eluates were separated by SDS (sodium dodecyl sulfate) electrophoresis and identified by mass-spectrometry (LC-MS/MS) after trypsin digestion as described previously^36^. In brief, the bands of interest were excised and in-gel digested in an adapted manner according to reference^37^. Gel pieces were washed 2 times by repeated addition and removing of 0.1 M NH_4_HCO_3_ and acetonitrile, respectively, followed by drying down in a vacuum centrifuge. The proteins were reduced by rehydrating the gel pieces in 10 mM dithiothreitol for 45 min at 56 °C and further carbamidomethylated by adding 55 mM iodineacetamide for 30 min at room temperature. Gel pieces were washed again 2 times, dried down, rehydrated by a freshly prepared digestion buffer containing 50 mM NH_4_HCO_3_ and 12.5 ng/μL of trypsin gold (Promega), and incubated at 37 °C overnight. Generated tryptic peptides were extracted from the gel by repeated addition of a sufficient volume of 25 mM NH_4_HCO_3_ and acetonitrile, respectively. The extraction was forced by sonication. All extracts were pooled and dried down in a vacuum centrifuge. The peptides were redissolved in 5 μl of 0.1% trifluoroacetic acid and purified on ZIP-TIP, C18-nanocolumns (Millipore, Billerica, USA). Peptides were eluted in 7 μl of 70% (v/v) acetonitrile and subsequently dried in a vacuum centrifuge. Dried samples were dissolved in 10 μl of 2% acetonitrile/0.1% trifluoroacetic acid and applied to an Ultimate 3000 Nano-HPLC (Dionex, Germany). Each sample was first trapped on a 1 mm PepMap-trapping column (Dionex, Germany) for 10 min at 30 μl/min of 2% acetonitrile/0.1% trifluoroacetic acid and subsequently subjected to a 75 μm inner diameter, 5 cm PepMap C18-column (Dionex, Germany). Peptide separation was performed by an acetonitrile-gradient (2%–50% acetonitrile for 40 min; 50%–90% acetonitrile for 10 min) at 300 nl/min. The separation column outlet was online coupled to a nano-spray interface (Bruker, Germany) of an Esquire HCT ETDII-Iontrap mass spectrometer (Bruker, Germany). Mass spectra were acquired in positive MS mode, tuned for tryptic peptides. MS/MS-precursor selection was performed in an optimized automatic regime, with preference for double and triple charged ions. Every selected precursor was fragmented by collision induced dissociation (CID) and electron transfer dissociation (ETD), respectively. MS/MS spectra were processed by the Data Analysis and BioTools software from Bruker, Germany. Combined CID/ETD-derived fragment lists were analyzed by two independent in-built statistical analysis algorithms: MASCOT probability scoring (Matrix Science Ltd, London, UK) and ProteinExtractor scoring (Bruker Daltonics, Bremen, Germany). Scores for peptide identification are given in Supplementary Table S1. The final table of the thiamin and thiazolium proteomes includes the best of repeated identifications of each protein.

### Bioinformatics approaches

Web-based bioinformatics resources DAVID (http://david.abcc.ncifcrf.gov)^38^; STRING (http://string-db.org/)^39^ and PANTHER (http://www.pantherdb.org/)^40^; were used to analyze the proteomes. PROSITE (http://prosite.expasy.org/)^41^ was used to create patterns^42^ and search for their matches in other proteins. Multiple alignments were done using Jalview (http://www.jalview.org/)^43^, CLUSTAL Omega^44^ and MUSCLE^45^. All manipulations with three dimensional (3D) structures were performed by PyMOL (http://www.pymol.org/)^46^.

### Identification of the thiamin-binding patterns in proteins

In order to localize potential thiamin binding sites in the proteins bound to the thiamin- and DMHT-modified sorbents, protein structural elements binding thiamin and its thiazole ring were identified from the solved structures of human thiamin diphosphokinase (UNIPROT_ID: TPK1_HUMAN) in complex with ThDP (PDB ID: 3S4Y) and the thiamin-synthesizing bacterial enzyme ThiM (UNIPROT_ID: THIM_BACSU) in complex with 4-methyl-5-(2-hydroxyethyl) thiazole (PDB ID: 1C3Q), respectively. A common structural element binding thiamin in human thiamin diphosphokinase and bacterial periplasmic thiamin/ThDP-binding protein (UNIPROT_ID: THIB_ECOLI)^47^ was found by examining their solved structures with ThDP and thiamin, PDB ID: 3S4Y and PDB ID: 2QRY, respectively.

Multiple sequence alignments of the pattern-providing enzymes from different organisms were then performed by CLUSTAL Omega^44^ and MUSCLE^45^ to find possible variations in the pattern structures. The resulting patterns [QK]x(0,1)Dx(0,1)[TS]Dx(3)[ACMVITS][LVIMF] and [ILV][ST][ST][ST]N (thiamin diphosphokinase- and thiamin diphosphokinase/ThiB-based), Ax(9)[PA][AVILFM][MI]x(20,22)[GA][TSNKAH] (ThiM-based), and the known motif common for the ThDP-dependent enzymes^48^, updated as G[DE][GA]x(24,30)NN according to the recent multiple sequence alignment^49^ were submitted to PROSITE (http://prosite.expasy.org/)^41^. Sequences of malate dehydrogenases, glutamate dehydrogenases and pyridoxal/pyridoxamine kinases found by the PROSITE scan against the thiamin-binding patterns were aligned to the orthologues with the resolved 3D structures, based on multiple sequence alignment of app. 70 proteins. The thiamin-binding patterns were visualized in the 3D structures of the proteins of interest using PyMOL (http://www.pymol.org/)^46^.

### Enzymes and assays

During purification of the thiamin phosphate hydrolase(s) from the synaptosomal protein fraction as described earlier^26^,^50^, routine assays of the eluate were done using the malachite green determination of phosphate^51^ released upon ThDP hydrolysis. Cytosolic fraction and extracts of brain mitochondria were used to assay brain enzymes, such as cytosolic and mitochondrial malate dehydrogenases and glutamate dehydrogenase. Brain homogenate fractionation and mitochondria sonication were done according to the published procedure^36^, with the mitochondrial pellet washed three times in order to ensure separation of the cytosolic enzymes. Wild-type human pyridoxal kinase (PdxK) was expressed and purified as previously published^52^, except for final dialysis that was carried out overnight against 50 mM sodium N,N-bis(2-hydrozyethyl)-2-aminoethanesulfonate (BES) buffer, pH 7.3. Varied conditions of the enzymatic assays are given in the figure legends. Assay of the enzyme fraction added to the medium omitting one substrate was used as a blank. The assays were mostly performed in the Ringer-bicarbonate buffer, pH 7.4, used for the protein binding to affinity sorbents. However, PdxK was inhibited by the ionic composition of this buffer, owing to which its activity was measured as described earlier^53^. Briefly, initial velocity studies for the conversion of pyridoxal to pyridoxal-5’-phosphate were followed at 388 nm in 50 mM sodium BES buffer, pH 7.3. Inhibition by thiamin and ThDP was studied at quasi-saturating concentrations of both substrates (1.8 mM of MgATP and 0.6 mM of pyridoxal) and increasing concentrations of inhibitors, ranging from 1 to 30 mM. Experimental data were fitted to the hyperbolic Equation (1) in which *Y* is the fractional activity, [I] is the concentration of inhibitor and 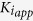 is the apparent inhibition constant:


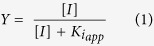


Kinetic parameters and inhibition constants were determined by Lineweaver-Burk analysis and corresponding secondary plots as summarized in^54^.

### Statistical analysis

Differences between the affected and control values were estimated by Student’s *t*-test. At least three replicates were used when assaying the enzyme activities.

## Results

### Affinity purification of synaptosomal thiamin and thiazolium proteomes

Parkhomenko *et al.* showed earlier that the proteins binding S^35^-thiamin and hydrolizing ThTP, ThDP, ThMP with the relative efficiencies 100%, 60% and 20%, correspondingly, are co-eluted from the affine sorbent modified with thiamin^14^,^15^,^17^,^26^. Our affinity purification was based on these studies. The procedure included a step-wise salt and urea elution from the affinity sorbents with thiamin or 3-decyloxycarbonylmethyl-4-methyl-5-(2-hydroxyethyl) thiazolium (DMHT) covalently bound to a spacer. Table 1 summarizes affinity purification of the acetone-delipidated fraction of synaptosomal proteins to obtain the thiamin-binding proteins containing the thiamin phosphates hydrolase activity (ThDPase, Table 1). The total protein portion of the acetone-delipidated fraction represented about 0.4% of the tissue fresh weight. Combined (i.e. fractions eluted with 1 M NaCl and 2 M urea) yield of protein from the affinity thiamin- and DMHT-modified sorbents was 2.7 and 1.6% of the protein applied to the two sorbents, respectively. SDS-electrophoresis of the pooled fractions containing the hydrolase activity towards thiamin phosphates in the eluates from the two sorbents is shown in Supplementary Fig. S1. As seen from Table 1 and Supplementary Fig. S1, 1 M NaCl efficiently disrupted the protein interactions with thiamin-modified sorbent, whereas 2 M urea was a better eluent from the DMHT-modified sorbent, probably due to additional hydrophobic interaction with the decyl moiety of DMHT. MS identification of eluted proteins (Supplementary Table S1) showed that there was no clear distribution of proteins eluted from the same sorbent by NaCl and urea. That is, the same proteins could elute either with NaCl or urea from the thiamin- or DMHT-modified sorbents. Therefore, the combined proteome (i.e. eluted from each sorbent by both NaCl and urea) was further analyzed.

### Overall analysis of the proteomes eluted from the thiamin- and DMHT-modified affinity sorbents

Specific binding of proteins to affinity sorbents may occur through (i) direct interaction with the bait and (ii) interaction of the bait with biologically relevant heterologous protein-protein complex, given that one protein of the complex specifically interacts with the bait. Table 2 shows that the proteomes eluted from the two sorbents contained many proteins known to bind thiamin compounds and interaction partners of such proteins. Taking into account the total number of identified proteins (150 and 57 in the thiazolium and thiamin proteomes, respectively, (Supplementary Table S1), the proteins known to bind thiamin and their immediate interaction partners (Table 2) accounted for 10–13% of the proteomes. In view of the fact that heterologous complexes may involve not only the known and direct partners of the thiamin-dependent proteins shown in Table 2, but also those unknown and indirect, this value corresponds to the lowest limit of specific interactions with the affinity sorbents. In addition to the interactions listed in Table 2, multiple indirect or unknown interactions with thiamin are supported by published data. In particular, the thiazolium proteome (Supplementary Table S1) includes the protein PHYIP_RAT, annotated as interacting with phytanoyl-CoA hydroxylase, the first enzyme of the ThDP-dependent pathway of phytanoyl-CoA degradation^55^. Furthermore, many proteins listed in Supplementary Table S1 were shown to co-immunoprecipitate with ThDP-dependent 2-oxoglutarate dehydrogenase (OGDH) in an independent study which by molecular biology approaches revealed the interaction of OGDH with fatty acid metabolism^56^. Thiazolium proteome also includes DJ-1 protein whose orthologs in a variety of eukaryotic species are closely related to bacterial ThiJ kinases involved in the biosynthesis of thiamin^57^. Aldose reductase ALDR_RAT and glyoxalase II GLO2_RAT (present in both thiamin and thiazolium proteomes; Supplementary Table S1) detoxify glyoxal and methylglyoxal, which increase upon addition of thiamin antagonist, oxythiamin^58^, or in thiamin-deficient animals^59^. *In vitro* assays of tissue homogenates from such animals showed that the glyoxalase activity decreased in thiamin deficiency and increased upon thiamin repletion^59^, similarly to the activities of ThDP-dependent enzymes. In view of the presence of glyoxalase in the eluates from thiamin and DMHT-modified sorbents, these data favor the glyoxalase activation by thiamin or derivatives, and inactivation by the thiamin antagonist oxythiamin. Functional relationship of the ThDP-dependent 2-oxoglutarate dehydrogenase to glutamate and γ-aminobutyric acid (GABA)^60^,^61^ signaling and metabolism correlates with the presence of the glutamate receptor 2 and enzymes belonging to the glutamate and GABA-related pathways in the proteomes, such as mitochondrial and cytoplasmic aspartate aminotransferases, glutamine synthetase, succinate-semialdehyde dehydrogenase, 4-aminobutyrate aminotransferase (Supplementary Table S1). Published data also reveal a number of functional and physical interactions between the proteins eluted from the thiamin and/or DMHT-modified sorbents (Table 3). Partial overlapping of the proteins shown in Tables 2 and 3 includes HSP-70 and enzymes of the tricarboxylic acid (TCA) cycle with its associated pathways. These identified proteins may hence be the core components of extended protein-protein interacting structures.

**Figure 1:**
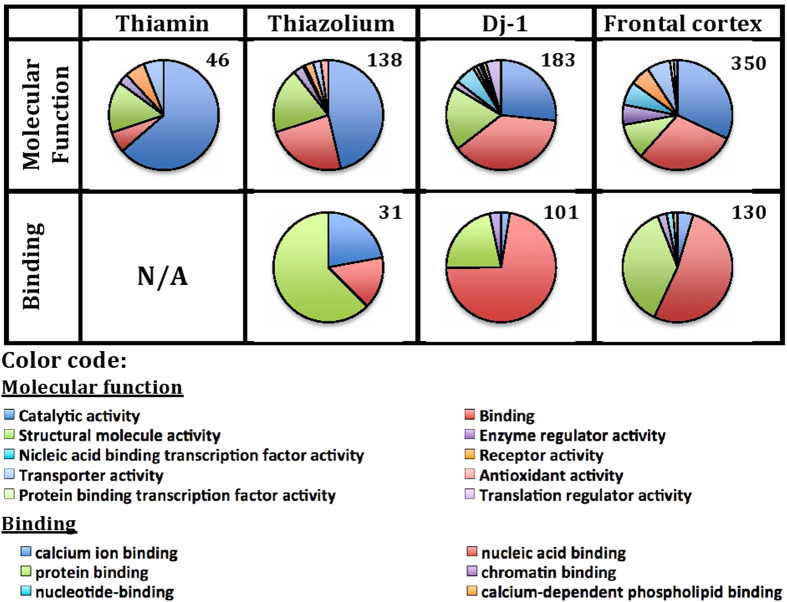
Classification by PANTHER of proteins of the thiamin, thiazolium, DJ-1-binding^32^ and frontal cortex^33^ proteomes according to their molecular functions and binding properties. Color code of the circle diagram sections are shown in the figure.

In addition to our own analysis of the proteomes according to a strict criterion of experimentally confirmed biologically relevant interactions (Tables 2 and 3), we performed automatic database search using bioinformatics tools. Supplementary Fig. S2 visualizes different types of interactions between proteins of the identified proteomes extracted from the databases by STRING. High interaction density defines clusters of proteins involved not only in the expected ThDP-dependent metabolism (including that of glutamate; blue circle), but also in signaling through 14-3-3 (red circle). Additional clusters of proteins involved in cellular redox state homeostasis (green circle) and Ca^2+^ regulation (yellow circle) were revealed in the thiazolium proteome (Supplementary Fig. S2B), where more proteins were eluted and identified (Supplementary Fig. S1 and Table S1). A further study assessed functional classification of the proteins in the thiamin and thiazolium proteomes by DAVID and PANTHER. In order to exclude potential bias due to overrepresentation of widely studied phenomena (such as, e.g. regulation of proteins by phosphorylation, contributing to the term “phosphoprotein”) we used as a reference the published partial brain proteomes of DJ-1-binding proteins^32^ and frontal cortex^33^. Functional annotation by DAVID (Table 4) revealed that the thiamin and thiazolium proteomes are highly enriched with acetylation-related proteins, suggesting an interplay between this newly emerged regulatory post-translational modification and thiamin. The same annotation term is highly enriched in the proteome binding to the PD-associated protein, DJ-1 (parkin-7)^32^, which is present in the thiazolium proteome (Supplementary Table S1). However, proteome of frontal cortex^33^ is mostly enriched with another annotation term, i.e. phosphoprotein, thus supporting specific association of the proteins eluted from the thiamin- and DMHT-modified sorbents with acetylation. Nevertheless, phosphoproteins also comprise a significant part of the thiamin and thiazolium proteomes, which agrees with the known ThTP-dependent phosphorylation of synaptosomal proteins^18^. However, there is more than a 2-fold difference in orders of magnitude of *P*-values between the first and second most abundant (i.e., with high *n*) groups of proteins (Table 4). In other words, the new relation of the thiamin and thiazolium proteomes to acetylation revealed in this work has much more statistical significance compared to other terms. Functional classification by PANTHER indicated three major functions of the thiamin- and thiazolium-binding proteins: *catalytic activity*, *binding* and *structural molecule* *activity* (Fig. 1, Molecular function). The binding activity includes proteins involved in Ca^2+^, nucleic acids binding and protein-protein interactions; the structural molecule activity includes structural proteins of a cell, such as structural constituents of cytoskeleton and ribosomes. Once again, the proteome specificity of the classification is obvious from comparative analysis of the thiamin/thiazolium and other proteomes. For example, protein binding function is dominating in the thiazolium proteome, whereas nucleic acid binding is the major term in DJ-1 and frontal cortex proteomes (Fig. 1, Binding).

### Kinetic proof of the thiamin and DMHT binding to abundant enzymes of the thiamin and thiazolium proteomes

Other then known thiamin or ThDP-dependent proteins (exemplified in Table 2), we were able to detect the interactions with the thiamin and DMHT baits of proteins unknown to be thiamin-dependent. High abundance of a protein in the affinity chromatography eluate suggests the direct, rather than protein-mediated, interaction of the protein with the bait. Abundance of a protein is roughly proportional to the number of identified peptides and sequence coverage, correlating with high score values of the protein identification^36^. The reactivity of the most abundant enzymes towards thiamin and DMHT was therefore studied by means of enzyme kinetics. Changes of enzymatic reaction rates in the presence of the compounds of interest are sensitive and efficient indicators of the compound binding to enzyme. Owing to this, independently of potential biological significance of the binding and amplitude of the changes, effects of thiamin and DMHT on the activity of the most abundant enzymes in the proteomes should prove their direct interaction with the baits. Because the efficiency and effects of ligand binding to the enzymes may be strongly affected by medium conditions, the primary choice for the enzymatic assays was Ringer-bicarbonate buffer, used for protein application to the affinity columns. This allowed us to mimic conditions of potential protein interaction with the affinity baits.

**Figure 2:**
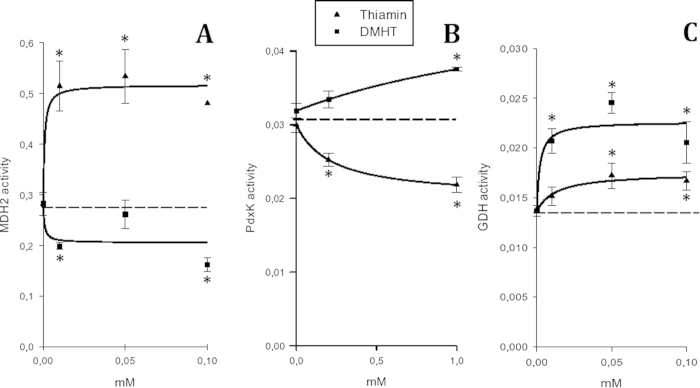
Influence of thiamin and DMHT on activities of the enzymes abundant in the thiamin and thiazolium proteomes. (**A**)—mitochondrial malate dehydrogenase (MDH2) assay in Ringer-bicarbonate buffer, pH 7.4, at 0.01 mM oxaloacetate and 0.02 mM NADH; (**B**)—human recombinant pyridoxal kinase (PdxK) assay in 75 mM NaBES, pH 7.3, at 0.125 mM pyridoxal and 0.1 mM ATP; (**C**)—glutamate dehydrogenase (GDH) assay in Ringer-bicarbonate buffer, pH 7.4, at 0.1 mM 2-oxoglutarate and 0.02 mM NADH. Activities are expressed in micromoles of substrate transformed per min per mg of protein. Each data point represents the average ± SEM from at least triplicate assays. When error bars are not visible, they are within the symbol size. The experimental curves were approximated by hyperbolic functions using SigmaPlot 12.0. Statistical significance (p ≤ 0.05, t-test) of the differences compared to the control values is marked by asterisks.

As seen from Supplementary Table S1, mitochondrial malate dehydrogenase (MDH2) was identified in the thiamin proteome with the highest confidence score and peptide number; in the thiazolium proteome, it occupied the second place after the known thiamin ligand albumin (Table 2). Direct interaction of MDH2 with both the thiamin and DMHT baits was supported by kinetic assays of MDH2 in the extracts of brain mitochondria. As seen in Fig. 2A, thiamin and DMHT affected enzyme activity starting from concentrations as low as 10 μM. Remarkably, a 2-fold activation by thiamin was not mimicked by its structural analog DMHT, yet binding of the latter was obvious from its inhibitory effect on MDH2 (Fig. 2A). The dependence of enzymatic response, such as activation or inhibition, on the effector structure is a known feature of allosteric regulation.

Pyridoxal kinase (PdxK) was identified in the thiamin proteome with the next highest confidence score after MDH2. In the thiazolium proteome, the enzyme was also identified with high score and peptide number, following MDH2 after abundant cellular proteins forming multiple protein-protein interactions, such as actin, tubulin and 14-3-3 proteins (Supplementary Table S1). Because high concentrations of Mg^2+^ and Ca^2+^ in the Ringer-bicarbonate buffer inhibit recombinant human PdxK, its reactivity with thiamin and DMHT was assayed under standard conditions established earlier^53^. Figure 2B shows that both compounds affected PdxK activity, supporting the direct interaction of the enzyme with the corresponding baits of the affinity sorbents. The low affinity of the thiamin compounds to PdxK (10^−4^–10^−3^ M) in the kinetic assays, compared to the other tested enzymes (i.e. malate and glutamate dehydrogenases; 10^−5^–10^−4^ M) could be due to specific post-translational modifications of brain PdxK, not occurring in the recombinant enzyme used in our kinetic studies. Because detailed enzymological characterization of recombinant human PdxK was far beyond the scope of the present study, we limited our kinetic studies of PdxK to the model system established earlier for recombinant human enzyme^53^.

Mitochondrial glutamate dehydrogenase (GDH) was identified with high score as an abundant enzyme in the thiazolium, but not thiamin proteome (Supplementary Table S1). Indeed, GDH activity assays in Ringer-bicarbonate buffer indicated that the effect of DMHT was of higher amplitude compared to that of thiamin (Fig. 2C). Hence, it is probably not thiamin, but some of its natural derivatives that bind at the DMHT site. In any case, the kinetic data presented in Fig. 2C are in good accord with the identification of GDH in the thiazolium proteome only. Statistical significance of the effects shown in Fig. 2 was confirmed by reproducing the results with different enzyme preparations, including commercially available pure enzymes, and under variable sets of conditions, as presented in the following sections.

All together, our kinetic studies provide a proof of concept for the thiamin dependence of those proteins which were found abundant in the eluates from affinity sorbents.

### Localization of thiamin-binding sites in MDH, GDH and pyridoxal kinase by structural and kinetic approaches

**Figure 3:**
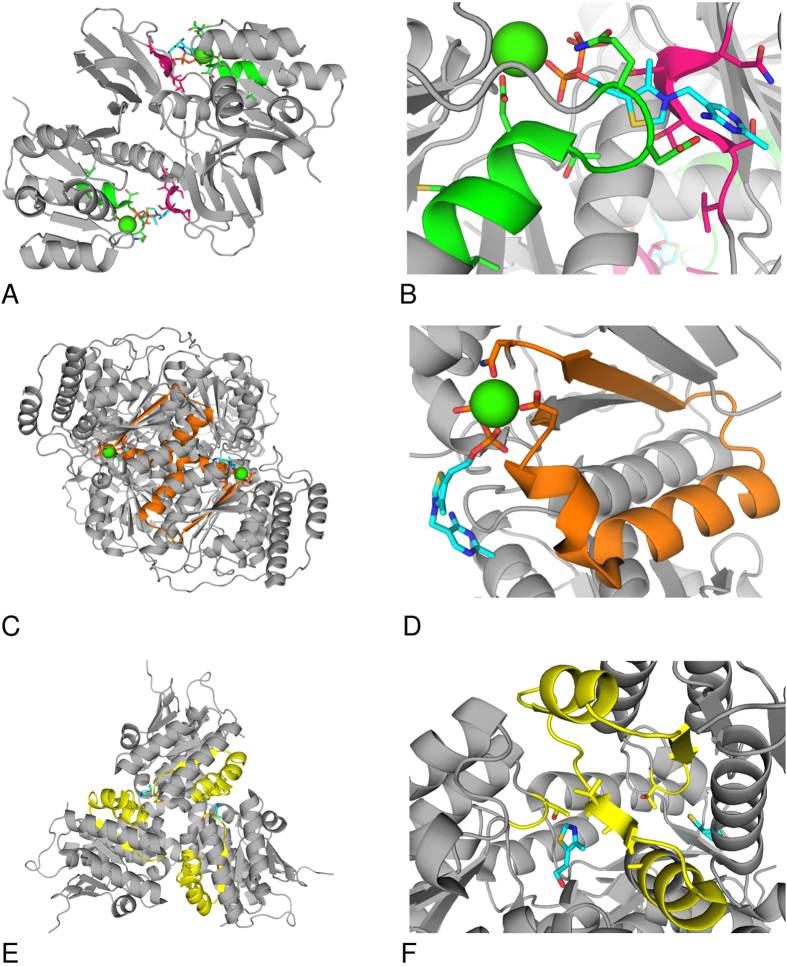
Patterns for binding of thiamin and derivatives. **A**,**B**—ThDP binding patterns (marked in green and pink) in thiamin diphosphokinase PDB ID: 3S4Y (A—dimer, B—close view). **C**,**D**—ThDP-binding motif of ThDP-dependent enzymes (marked in orange) in transketolase (C—dimer, PDB ID: 3OOY, D—close view, PDB ID: 3MOS). **E**,**F**—Binding pattern of 4-methyl-5-(2-hydroxyethyl)-thiazole (marked in yellow) in ThiM PDB ID: 1C3Q (E—trimer, F—close view). Essential residues of the patterns are shown as sticks in the same color as the pattern. The active site metal ions are shown in green; other ligands are in cyan; heteroatoms are presented according to the common color code: red for oxygen, blue for nitrogen, yellow for sulfur, orange for phosphorus.

In order to localize potential binding sites for thiamin or its derivatives in the enzymes shown to directly interact with thiamin and DMHT in kinetic studies (Fig. 2), we made advantages of available structural information on binding sites for thiamin and derivatives^23^ and on the solved 3D structures of MDH, GDH and pyridoxal kinase. Because all these enzymes interacted with DMHT (Fig. 2), which mimics the thiazolium part of thiamin, the binding patterns were generated using the structures of thiamin- and thiazol-dependent enzymes, such as human thiamin diphosphokinase and bacterial 4-methyl-5-(2-hydroxyethyl)-thiazole kinase (ThiM). In addition, the pattern based on the known characteristic motif of the ThDP-dependent enzymes^48^ was employed for the PROSITE search of protein sequences with potential thiamin-binding sites. When creating the binding patterns for thiamin and derivatives in the enzymes (as described in Methods) we were also interested in finding common and thiamin-specific elements involved in binding. In this regard, the protein residues in the vicinity of thiamin heterocycles were considered more promising, because the phosphate groups are present in many other compounds. Examination of different protein complexes with thiamin and derivatives pointed to a high occurrence of serine and threonine residues neighboring the thiazolium ring of thiamin. In particular, the bacterial thiamin-binding periplasmic protein ThiB (synonyms tbpA, yabL) which is a part of the thiamin ABC (ATP-binding cassette) transporter (the complex responsible for the uptake of thiamin and its phosphate derivatives^47^) exhibited a stretch of serine residues whose conformation and position relative to the thiamin heterocycles were highly similar to those found in thiamin diphosphokinase. We used this motif (Fig. 3B) to construct the pattern [ILV][ST][ST][ST]N which was found to be present in our proteins of interest. Furthermore, we shall refer to this hybrid pattern as the thiamin diphosphokinase/ThiB pattern, in order to distinguish it from the other one based solely on thiamin diphosphokinase.

**Figure 4:**
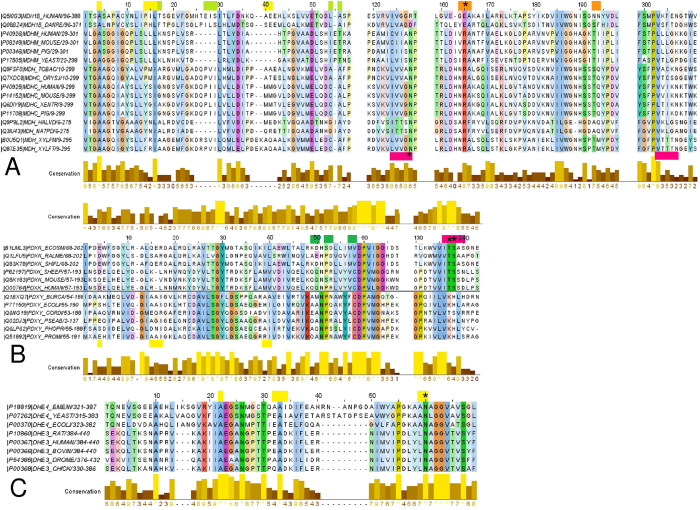
Local multiple sequence alignments of sequence parts comprising the thiamin and derivatives-binding patterns found in the abundant enzymes of the thiamin and thiazolium proteomes. (**A**)—malate dehydrogenases; (**B**)—pyridoxal kinases; (**C**)—glutamate dehydrogenases. The pattern residues are marked above or below the sequences dependent on the vicinity of the sequences where they are found. The pattern color code is as in Fig. 3, except for the two PROSITE hits to thiazole-binding pattern of ThiM in MDH (**A**), where the yellow-green color marks the second hit to the pattern. The pattern residues common with those of the active sites are marked by asterisks. The conservation is given below the alignments in (**A** and **C**). In (**B**), the conservation above and below the alignment corresponds to PdxK and PdxY, present in the upper and lower parts of the alignment, correspondingly. Protein IDs and accession codes, according to UNIPROT database, precede the alignments.

**Figure 5:**
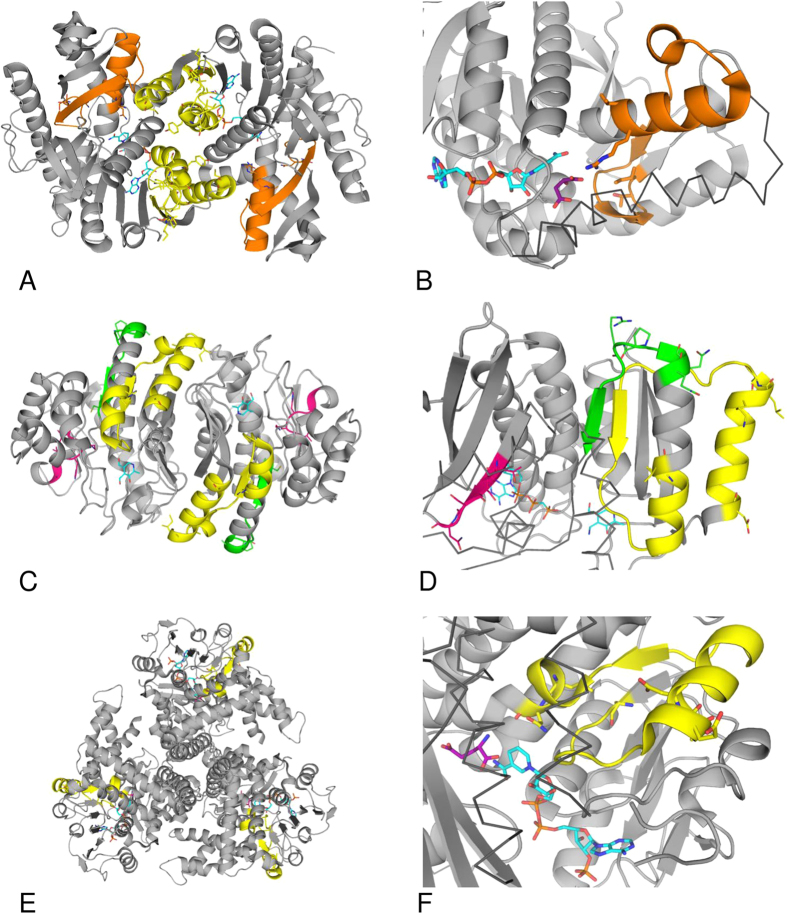
Thiamin and derivatives binding patterns in the 3D structures of the abundant enzymes of thiamin and thiazolium proteomes. The pattern color code and other details are as in Fig. 3. (**A**)—Dimer of porcine cytosolic MDH1 with NAD^+^ bound (PDB ID: 4MDH). (**B**)—Close view of the ThDP-binding motif of ThDP enzymes in the monomer of human mitochondrial MDH2 with NAD^+^ and D-malate in the active site (PDB ID: 2DFD). (**C**)—Dimer of *E. coli* PdxY with pyridoxal bound to the active site (PDB ID: 1TD2). (**D**)—Close view of the thiazole and thiamin-binding patterns in the monomer of sheep PdxK with bound pyridoxamine and phosphomethylphosphonic acid adenylate ester (PDB ID: 1RFT). (**E**)—Trimer of bovine GDH with NADPH and glutamate bound to the active sites (PDB ID: 3MVQ). (**F**)—Close view of the thiazole-binding pattern of ThiM near the active site of bovine GDH with NADPH and glutamate bound (PDB ID: 3MVQ).

Figure 3 shows the four employed patterns, i.e. the thiamin diphosphokinase- and thiamin diphosphokinase/ThiB-based patterns (A, B), the pattern present in ThDP-dependent enzymes (C, D) and the ThiM-based pattern (E, F), in the corresponding enzyme complexes with ThDP or thiazole, both in oligomeric structures and at a close view. The proteins relevant to this study, i.e. those shown to interact with thiamin and DMHT by both affinity chromatography and kinetics, were selected among the PROSITE hits to the patterns. The thiamin-binding pattern of thiamin diphosphokinase was found in bacterial pyridoxal kinase PDXK_ECOLSM. The hybrid thiamin diphosphokinase/ThiB-based pattern matched to animal pyridoxal kinase PDXK_SHEEP, fungal glutamate dehydrogenase DHE2_ACHKL and 10 malate dehydrogenases, including four bacterial, five archaeal and one eukaryotic (MDH1B_DANRE) species. The thiazole-binding pattern of ThiM was found within 13 sequences of pyridoxal kinase isoform PdxY (from different *Burkholderia* and *Pseudomonas* strains), three sequences of bacterial and fungal glutamate dehydrogenases (DHE2_ACHKL), and 36 sequences of malate dehydrogenases from bacteria and plants. The ThDP-binding motif matched to the different parts of sequences from MDH1B_HUMAN and MDH_DESRM. The pattern-comprising parts of the alignments of the thiamin/DMHT-binding enzymes are given in Fig. 4. Using multiple sequence alignments, the patterns were identified in the 3D protein structures as shown in Fig. 5. Available 3D structures of the enzymes which in our study were shown to bind thiamin and DMHT, included porcine cytosolic MDH1 (PDB ID: 4MDH), human mitochondrial MDH2 (PDB ID: 2DFD), archeal MDH (PDB ID: 2 × 0R), bacterial MDH (PDB ID: 1B8U), PdxK from sheep (PDB ID: 1RFT), the pyridoxal kinase isoform PdxY from *E. coli* 62 (PDB ID: 1TD2), and bovine GDH (PDB ID: 3MVQ).

**Figure 6:**
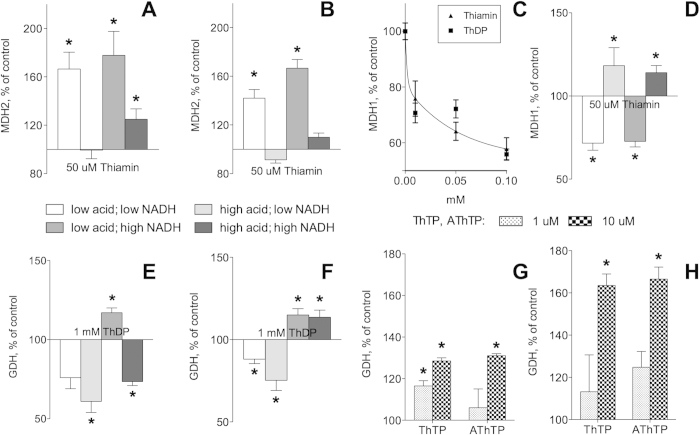
Regulatory effects of thiamin and derivatives on partially isolated rat brain enzymes MDH2 (**A**), MDH1 (**C**,**D**), GDH (**E**,**G**) and purified porcine mitochondrial malate dehydrogenase (**B**) and bovine liver GDH (**F**,**H**). ThDP—thiamin diphosphate; ThTP—thiamin triphosphate; AThTP—adenylated thiamin triphosphate. (**A**,**B**,**D**–**F**)—Dependence on the substrate saturation. (**C**,**G**,**H**)—Dependence on concentrations of thiamin and derivatives. Shades of grey define different saturations with the dicarboxylic acid substrate (i.e. oxaloacetate or 2-oxoglutarate (2-OG), generically referred as “acid” in the figure) and NADH as indicated in the common legend to the figures (**A**,**B**,**D**–**F**). Bar patterns define varying concentrations of ThTP and AThTP as shown in the common legend to the figures (**G** and **H**). The enzymes were assayed in Ringer-bicarbonate (**A**–**D**,**G**,**H**) or 100 mM Tris-HCl buffers (**E**,**F**), pH 7.5, using the following substrate concentrations: (**A**,**B** and **D**)—0.01 mM oxaloacetate (low acid); 0.3 mM oxaloacetate (high acid); 0.02 mM NADH (low NADH); 0.14 mM NADH (high NADH); (**C**)—0.01 mM oxaloacetate, 0.02 mM NADH; (**E**,**F**)—0.1 mM 2-oxoglutarate (low acid), 2.5 mM 2-OG (high acid); 0.02 mM NADH (low NADH); 0.2 mM NADH (high NADH); **G**,**H**—0.1 mM 2-OG, 0.2 mM NADH.

The patterns in the PROSITE-predicted protein hits to the enzymes which were shown to bind thiamin and DMHT (Fig. 5) were compared to the original patterns (Fig. 3). We considered the preservation of the 3D pattern conformation, the conservation of the pattern-comprising protein structure and co-localization of different patterns in one enzyme as enhancing the probability that the pattern belongs to a functional thiamin-binding site (Fig. 2). In particular, using multiple sequence alignment and the PDB ID 1IB6 structure of *E. coli* MDH, we found that spatial conformation of the motif in MDH_DESRM differs from the original one presented in Fig. 3D, not supporting functional significance of the motif found in MDH_DESRM. The same applied to the thiamin diphosphokinase/ThiB pattern in the sequence of MDH1B_DANRE (residues 157–161). In the available 3D structures of MDH the pattern residues are found within a α-helix instead of the β-sheet present in the original pattern (Fig. 3B). In contrast, the ThDP-binding motif found in the putative and still uncharacterized human isoform MDH1B (Fig. 4) not only had an appropriate conformation (as shown in Fig. 5A,B), but also co-localized with the two thiazole-binding patterns found in other MDH sequences (Figs 4 and 5). The exclusive presence of the ThDP-binding motif in no other MDH but MDH1B (Fig. 4A), was due to the lack of significant residues of the motif in the two known isoforms of MDH. The motif G[DE][GA]x(24,30)NN bind Mg^2+^ forming a bridge to the diphosphate group of ThDP (Fig. 3D). Moreover, the essential acidic residue of the G[DE][GA] triplet of the motif was substituted, in the known MDH isoforms, by a conserved arginine residue (Fig. 4A) interacting with malate (Fig. 5B). Thus, the ThDP-binding motif may bind Mg^2+^ in the uncharacterized MDH1B isoform but not in the known isoforms of cytosolic and mitochondrial MDHs. Nevertheless, kinetic studies on known MDH isoforms agree with their thiamin binding site near the malate/oxaloacetate binding site, i.e. near the site having the conserved arginine residue in common with the apparently different ThDP-binding motif (Fig. 5B). As shown in Fig. 6A, the thiamin-dependent activation of mitochondrial MDH2 disappeared upon saturation with oxaloacetate, but not upon saturation with NADH. Similar data were obtained using commercial preparation of mitochondrial MDH purified from porcine heart (Fig. 6B). Because thiamin stimulates the MDH2 reaction at a low saturation with oxaloacetate only, the effect is obviously due to increased affinity of the enzyme-thiamin complex to oxaloacetate. The kinetic data thus agree well with an allosteric site for thiamin near the oxaloacetate/malate site, pinpointed by the ThDP-binding motif (Fig. 5B). In the dimer structure, additional residues for the thiamin binding to mitochondrial MDH2 could be provided by the proximal thiazole-binding pattern of the neighboring subunit (Fig. 5A). The hits to this pattern were found by PROSITE in cytosolic MDH1 only, because mitochondrial MDH2 has organelle-specific sequence deletions in this region (Fig. 4A). However, the lacking third part of the pattern (AA duplet marked in yellow, Fig. 4A) was found in the mitochondrial sequence several residues later (AA before the conserved D, Fig. 4A), suggesting a complete thiazole-binding pattern in mitochondrial MDH2 as well.

Interestingly, the thiamin diphosphokinase/ThiB-based β-strand with the essential triplet of S/T residues was revealed in the two positions of the sequences in some bacterial and archaeal MDHs (Fig. 4A). Supplementary Fig. S3A shows that in archaeal MDH the thiamin-binding pattern localized close to the NAD^+^/malate binding site. In bacterial MDH (Supplementary Fig. S3B) the pattern belongs to β-strand which covers the NAD^+^-binding site, separating it from the solvent. Thus, although different thiamin-binding patterns could be found in different sequences of MDH, they all co-localize in the protein 3D structure near the enzyme active site.

The finding of the thiazole-binding pattern in cytosolic MDH1 forecasts the interaction of thiamin with this isoform too. As shown in Fig. 6C, cytosolic MDH showed a strong inhibition by both thiamin and ThDP, suggesting the diphosphate group to be not essential for the inhibitory effect. The thiamin-dependent inhibition of cytosolic MDH1 and the activation of the mitochondrial isoform (Figs 2A,6A) agree with the structural differences of the enzyme sequences in the regions where the ThDP-binding motif and thiazole-binding pattern were found (Fig. 4A). However, also in the cytosolic isoform the thiamin effect was dependent on saturation with oxaloacetate (Fig. 6D), in agreement with partial overlapping between ThDP-binding motif and oxaloacetate/malate-binding site (Fig. 5B).

**Figure 7:**
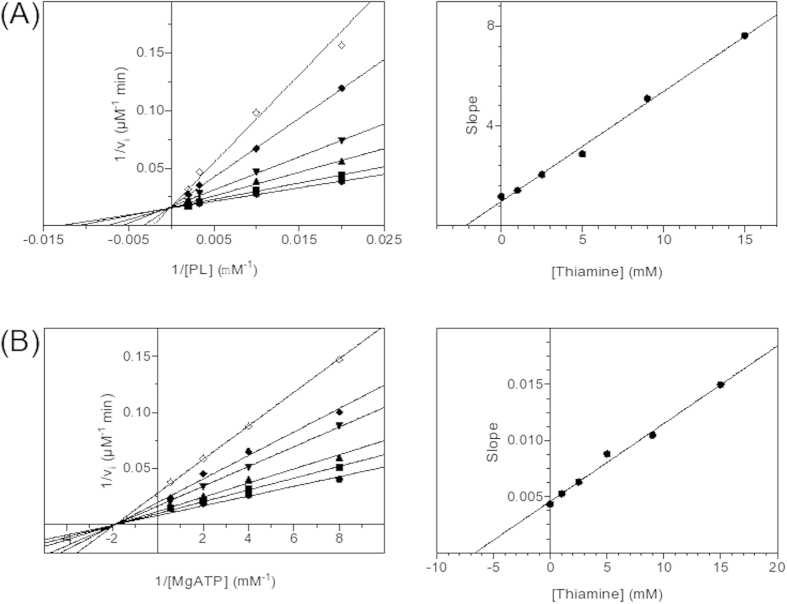
Kinetic analysis of human pyridoxal kinase inhibition by thiamin at varied substrate pyridoxal (**A**) or ATP (**B**) concentrations, using Lineweaver-Burk (left panel) and secondary plots (right panel). The thiamin concentrations in the Lineweaver-Burk plots are: • 0 mM, ■ 1.0 mM, ▲ 2.5 mM, ▼ 5.0 mM, ♦ 9.0 mM and ◊ 15 mM. All parameters are the average of two or three independent determinations, with SD less than ± 5%.

**Figure 8:**
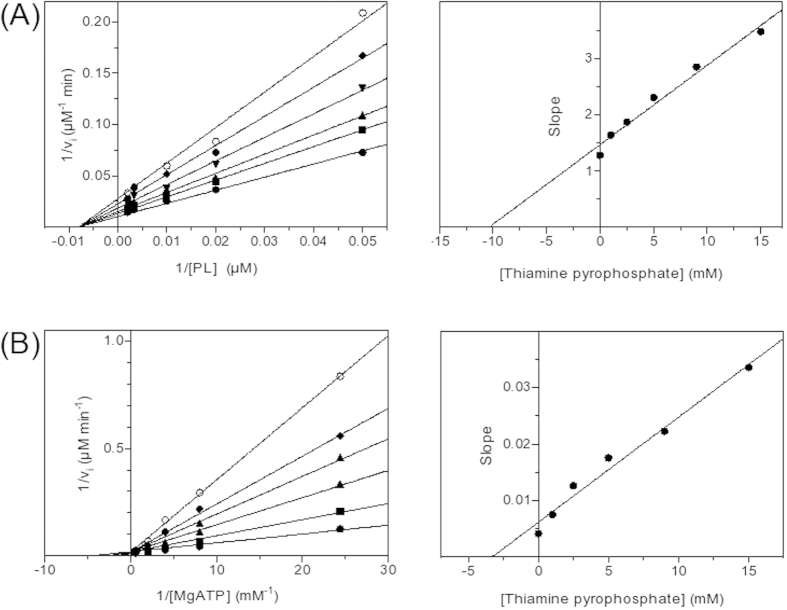
Kinetic analysis of human PdxK inhibition by thiamin diphosphate at varied pyridoxal (**A**) or ATP (**B**) concentrations, using Lineweaver-Burk (left panel) and secondary plots (right panel) The thiamin concentrations in the Lineweaver-Burk plots are: • 0 mM, ■ 1.0 mM, ▲ 2.5 mM, ▼ 5.0 mM, ♦ 9.0 mM and ◊ 15 mM. The points present average values of two or three independent determinations, with the SD less than ± 5%.

As mentioned above, PdxK from *E. coli* and PdxK from sheep (Fig. 4B) were shown to possess the thiamin-binding patterns based on thiamin diphosphokinase only (green, Figs 3A,B and 4B) or together with ThiB (pink, Figs 3A,B and 4B). The thiazole-binding pattern (yellow in Figs 3E,F and 4B) was found in an isoform of pyridoxal kinase existing in bacteria (PdxY). Although the two isoforms exhibit high sequence, structure and function similarities^63^, their sequence alignment in the pattern-comprising regions, i.e. the local consensus in these regions, favors different binding of the thiamin compounds to the isoforms (Figs 4B and 5C,D). Obviously, this is the reason of the differential PROSITE mapping of the patterns to the isoforms, as mentioned above. Along with the isoform-specific conservation of the essential residues of the patterns, the patterns have different localization in 3D structures. As seen from Fig. 5C,D, the thiamin-binding residues of the thiamin diphosphokinase- (green, better correspondent to PdxK) and ThiM-based (yellow, better correspondent to PdxY) patterns occupy positions distal from the active site. Owing to this, both patterns would define an allosteric binding site for thiamin or derivatives. The site is likely to communicate with the pyridoxal-binding site through the protein structure. In particular, the β-sheets (green and yellow) and a loop (yellow) distant from the thiamin binding residues of the patterns are in the vicinity of the substrate pyridoxal (Fig. 5D), and may thus transfer a signal between the thiamin- and pyridoxal-binding sites. The information transfer between the sites may also involve subunit interface close to the pattern residues (Fig. 5C). In contrast, the thiamin diphosphokinase/ThiB (pink) pattern found in PdxK only (Fig. 4B), localizes close to the ATP-binding site (Fig. 5C,D). As a result, in the PdxY isoform found in bacteria, structural data suggest an allosteric site for thiamin interacting with the pyridoxal site. In the PdxK isoform, found in both bacteria and mammals, also a second site for thiamin binding, which is near to the ATP site, is suggested by the thiamin diphosphokinase/ThiB pattern (pink, Fig. 5C,D). Kinetic study using the model system with homogeneous recombinant human PdxK is in good accordance with the structural hints for the two thiamin/derivative-binding sites in PdxK. As seen from Fig. 7, thiamin is competitive towards pyridoxal and non-competitive towards ATP. In contrast, ThDP is non-competitive towards pyridoxal but competitive towards co-substrate ATP (Fig. 8). Although the relative inhibition constants for thiamin and ThDP (Table 5) are too high to be physiologically relevant, this is most probably due to the reasons discussed above, such as the lack of post-translational modifications in recombinant human PdxK or the absence of specific ligands in the assay medium. Nevertheless, the kinetic study defines the different mechanisms of inhibition of PdxK by thiamin or ThDP. While thiamin rather affects the pyridoxal binding, ThDP interferes with binding ATP (Table 5). This agrees with the two binding sites for thiamin compounds in PdxK, as suggested by localization of the thiamin-binding patterns in the 3D structure on Fig. 5C,D. The ATP-binding site may partially overlap with the ThDP binding site through the residues binding the diphosphate group present in the two compounds. Structural difference between thiamin and pyridoxal favor the allosteric binding of thiamin at a separate site, decreasing the affinity to pyridoxal (Table 5) through information exchange between the allosteric and active sites (Fig. 5D) as discussed above.

The PROSITE scan against the thiazole-binding pattern of ThiM revealed three GDH hits: DHE4_EMENI, DHE2_PSEAE and DHE2_ACHKL. DHE2_ACHKL was also found when searched using the thiamin diphosphokinase/ThiB-based pattern. However, DHE2_PSEAE and DHE2_ACHKL, do not align with mammalian GDHs, and the 3D structures for these DHE2 are not available. Owing to this, the PROSITE hits to these of archeael and bacterial glutamate dehydrogenases could not be taken into account for analysis of the pattern localizations. Nevertheless, the existence of the thiamin-binding patterns in different types of GDHs favors physiological significance of potential GDH regulation by thiamin compounds. In contrast, the eukaryotic enzyme from *Aspergillus nidulans* (DHE4_EMENI) did align to mammalian GDHs. The alignment (Fig. 4C) shows that the absence of the PROSITE hit to the pattern in animal GDHs is due to a shorter linker between the essential residues of the pattern (residues between 40 and 50 of the alignment in Fig. 4C). With this exception, the pattern occupies a conserved part of the GDH sequence, and its essential residues are mostly conserved throughout species. It is worth noting that the original thiamin binding pattern in the ThiM trimer was formed by regions belonging to the neighboring subunits (Fig. 3E,F). In the trimer of GDH (PDB ID: 3MVQ) no such interaction between the patterns is observed (Fig. 5E). However, general structure of the pattern (helix-β-sheet-helix) is preserved, and many loops in the pattern-surrounding regions of GDH may provide additional residues to bind thiamin and/or its derivatives (Fig. 5F). Formation of the thiamin-binding site in GDH by the residues from a single subunit may explain shortage of the linker between the second and third parts of the pattern in animal GDH compared to the original ThiM-based pattern found in DHE4_EMENI (Fig. 4C). Overall, the residues of the thiazol-binding pattern in GDH form a plausible binding site, which is very close to those of NAD(P)H (3.25 Å) and glutamate/2-oxoglutarate (4.48 Å) (Fig. 5F). Moreover, the thiazole-binding pattern has one residue in common with the NAD(P)H-binding site (Fig. 4C, conserved N60 in the alignment). Results of kinetic study were in good accord with such localization of the thiamin-binding pattern. At low NADH saturation, 1 mM ThDP inhibited both the enzyme from brain mitochondrial extract and purified commercial GDH from bovine liver (Fig. 6E,F). At high NADH saturation, the inhibition was not observed with the purified GDH, further supporting partial overlapping of the ThDP and NADH binding sites on GDH. Instead, a slight activation, similar to the thiamin effect in Fig. 2C, was seen (Fig. 6F). With the brain enzyme from mitochondrial extract, more complex effects of ThDP were observed at high NADH, as the effects depended on the 2-oxoglutarate concentration (Fig. 6E). At low 2-oxoglutarate and high NADH concentration, a slight activation (within 20%) was observed at 1 mM ThDP concentration, similarly to that of the purified GDH. However, increasing 2-oxoglutarate and NADH concentration to saturating levels led to an inhibition effect at 1 mM ThDP concentration (Fig. 6E), which was not observed with the purified enzyme (Fig. 6F). The complexity of the ThDP-induced effects on GDH (Fig. 6E,F) suggests multiple binding modes of ThDP to GDH, similar to those revealed for the GDH regulation by phosphorylated nucleotides and dinucleotides, such as adenosine diphosphate (ADP), guanosine triphosphate (GTP) and NAD(P)H^64^. Dependent on concentration, all these diphosphate-comprising nucleotides could bind at the NAD(P)H, GTP and ADP sites of GDH. Owing to this, the difference between the purified and extracted GDH regarding the ThDP effect at saturation concentrations of both 2-oxoglutarate and NADH (Fig. 6E,F) could be due to a higher residual saturation of the non-purified mitochondrial enzyme with the GDH activator ADP. In the reaction medium, 1 mM ThDP could substitute endogenous ADP at the GDH activation site. If the allosteric activation by ADP cannot be mimicked by ThDP, the substitution would decrease the GDH activity due to decreased ADP activation. This assumption was supported by measuring the ThDP effect on the purified GDH at saturating NADH and 2-oxoglutarate concentrations in the presence of ADP. 2 mM ADP was shown to activate purified GDH about two-fold, with the activation abolished upon addition of 1 mM ThDP. Insofar, a residual saturation of non-purified mitochondrial GDH with endogenous ADP could be responsible for the difference between the purified GDH and the enzyme in mitochondrial extract regarding the ThDP effect at high saturation of NADH and 2-oxoglutarate (Fig. 6E,F). The residual saturation with endogenous regulators, along with tissue specificity of, e.g., posttranslational modifications, could also contribute to the different amplitudes of the effects in the mitochondrial and purified enzymes shown in Fig. 6.

Although both thiamin and ThDP affected GDH activity, the amplitudes of their effects and concentration dependence were higher when DMHT was taken into consideration (Fig. 2). The better binding of DMHT to GDH was obvious also from the GDH identification in the eluate from the DMHT-modified sorbent only (Supplementary Table S1). Moreover, both mitochondrial and purified GDH were shown to be activated in the presence of low concentrations (1–10 μM) of thiamin triphosphate and adenylated thiamin triphosphate (Fig. 6G,H), suggesting that the binding of thiamin, ThDP and DMHT partially mimics the GDH regulation by some other thiamin derivatives. High efficiency of the GDH activation by the above-mentioned non-coenzyme derivatives of thiamin points to GDH as an enzyme target of these compounds *in vivo*. A higher effect of the compounds on the purified GDH (Fig. 6H) compared to the enzyme in mitochondrial extract (Fig. 6G) simulates the difference of the ThDP effect on the two enzymes at saturating NADH concentration (Fig. 6E,F). Based on the existing^64^ and presented data (Fig. 6), a complex interaction of adenylated thiamin triphosphate and ThTP with the regulatory (ADP and GTP) and catalytic (NAD(P)H) nucleotide sites of GDH may be suggested, which certainly requires future studies.

## Discussion

### Novel targets of thiamin compounds in brain

To advance the understanding of molecular mechanisms of thiamin action in brain, we aimed at the identification of the synaptosomal proteins binding and/or transforming thiamin or its derivatives. The protocol of partial purification of a subset of such proteins included affinity chromatography on sorbents derivatized with thiamin or its thiazolium heterocycle covalently bound to a spacer. The approach was elaborated in the previous work to obtain a fraction of synaptosomal proteins which were able to bind labeled thiamin and hydrolyze thiamin phosphates, with thiamin triphosphate being the best substrate^13^,^26^. Affinity chromatography on sorbents carrying thiamin or its derivatives was earlier used to purify the thiamin- and ThDP-dependent enzymes, such as thiamin diphosphokinase^65^, pyruvate decarboxylase^35^ and pyruvate dehydrogenase^66^. In these studies, thiamin- and ThDP-dependent enzymes were not always successfully eluted by competitive desorption in the presence of thiamin or ThDP in the elution buffer. Most probably this was due to the protein conformational changes after the thiamin/ThDP binding, which have been structurally characterized in the recent years^22^. Because our work aimed at simultaneous identification of the unknown subset of synaptosomal thiamin- or thiamin derivative-binding proteins, an established protocol for the elution of such proteins in the presence of optimized salt and urea concentrations was preferred^14^,^15^,^17^,^26^. Besides a thiamin-modified sorbent, we also used a sorbent derivatized with the thiamin-specific thiazolium ring (DMHT-modified sorbent). This was done to reveal the proteins binding not only to the thiamin sorbent, but also to the sorbent including the unique thiazolium part of the thiamin molecule. The binding of the same proteins and/or protein clusters to these two sorbents was considered as evidence for the binding specificity to the thiazolium-possessing thiamin compounds. Interaction with the baits comprising the other parts of the thiamin structure, such as phosphates or aminopyrimidine residues, would not support the specificity, as the latter moieties or their analogs occur in abundant nucleotides interacting with many proteins. Besides, as specified in Introduction and discussed in details below, DMHT was shown to regulate neurotransmission. Moreover, the DMHT similarity with the thiamin degradation products could help fishing out potential targets of such products, which may acquire specific significance upon application of pharmacological doses of thiamin.

Whereas affinity chromatography aimed to purify single enzymes uses homogeneity criterion and enzyme specific activity to analyze the enzyme elution and the role of the bait structure, different strategies are employed in the high throughput approaches for isolation of a subset of proteins with their physiologically relevant yet unknown specific activities. In our study, we substantiate the specific binding of proteins to the baits through integration of results obtained by independent approaches. Our conclusion on the thiamin dependence of well-defined proteins or pathways does not solely rely on their binding to the thiamin- and DMHT-modified sorbents. The abundant proteins identified in the eluates from the affinity chromatography were independently tested for their interaction with the baits using enzyme kinetics and structural analysis. Besides, in view of emerging recognition of supramolecular structures in transmitting signals and organizing metabolism, we consider as biologically relevant not only the direct interactions of proteins with the baits, but also protein-mediated interactions. We revealed such interactions in our proteomes by both manual (Tables 2 and 3) and bioinformatics (Supplementary Fig. S2) analysis. The thiamin and thiazolium proteomes comprised many proteins (Supplementary Table S1, Fig. 1), clusters (Supplementary Fig. S2) and interactions (Tables 2 and 3, and Supplementary Fig. S2) which are shared by the two proteomes. The similarity provides a measure of the binding specificity in high throughput experiment, where it is not possible to check each protein regarding specificity of its interaction with the bait. As a result, affinity chromatography to isolate a subset of proteins generates leads for a further, more focused examination of the thiamin dependence of the attractive proteins or protein clusters identified in the eluates. In the current work, the specificity of the interaction with the thiamin and DMHT baits was proven for abundant enzymes of the proteomes by *in vitro* enzymatic assays and structural analysis. This study revealed mitochondrial and cytosolic malate dehydrogenases, glutamate dehydrogenase and pyridoxal kinase as novel targets of thiamin compounds in brain. These enzymes do not require ThDP as coenzyme. However, their saturation with the substrates (dicarboxylates, pyridoxal) structurally different from thiamin and/or its derivatives seems to be regulated by binding of thiamin compounds. The structural difference between substrates and effectors suggests allosteric binding sites, which are independently favored by our structural analysis. The observed effects on MDH, GDH and PdxK enzyme activity are highly dependent on the thiamin derivative used in the assay, on substrate saturation and medium conditions (Figs 2,6,7 and 8). Variations in the level of enzyme post-translational modifications and saturation with endogenous regulators may contribute to the quantitative differences between different enzyme preparations or between the enzymes in rat brain mitochondrial extract and purified commercial enzymes from other sources. However, significant activation of MDH2 (1.7-fold, Fig. 6A) and GDH (1.6-fold, Fig. 6F) by thiamin and derivatives is especially remarkable in view of the fact that unspecific effects and artifacts are usually inhibiting and rarely able to cause enzyme activation. Except for recombinant human PdxK, which might require a regulator and/or post-translational modifications to respond to physiological concentrations of thiamin and derivatives, affinity binding of the brain enzymes to the thiamin and DMHT baits was observed along with efficient regulation by the thiamin compounds at 10^−5^ M (Figs 2A,C and 6C,G,H). This concentration range is comparable to steady-state concentrations of total thiamin compounds (10^−5^ M of total thiamin and its phosphate derivatives; thiamin in particular 10^−6^ M), as determined in cell cultures and different tissues including brain^6^,^67^,^68^,^69^. It must be also taken into account that both intracellular compartmentalization and induced synthesis, such as observed for thiamin triphosphate and adenylated thiamin triphosphate^21^, may significantly increase temporary concentrations of different thiamin compounds in specific compartments. In particular, thiamin triphosphate and adenylated thiamin triphosphate have been recently shown to serve as alarmones, synthesized in response to amino acid and carbon starvation, although their protein targets were not identified^21^,^34^. Our data, indicating that GDH is a target of thiamin derivatives (Fig. 6G,H), agrees with earlier observation that thiamin triphosphate was synthesized *in vivo* under amino acid starvation only when cells oxidized pyruvate, but not malate^34^. It thus appears that the thiamin triphosphate-dependent regulation of GDH reaction involving a common intermediate of carbon and nitrogen metabolism, 2-oxoglutarate, is essential for metabolic checkpoint.

Overall, our analysis of the thiamin and thiazolium proteomes suggests that biologically relevant heterologous complexes, yet not known to be thiamin-dependent, may include proteins regulated by thiamin. That is, along with the known thiamin-binding proteins and their immediate interaction partners (Table 2), the eluates from the thiamin and DMHT baits also include proteins of specific metabolic and signaling pathways (Table 3) which may respond to thiamin through the interactions within supramolecular structures. The STRING-generated results (Supplementary Fig. S2) provided further support to the manual analysis of our data (Tables 2 and 3). Except for the signaling cluster of 14-3-3 proteins, all the STRING-identified clusters, such as those related to metabolism, reactive oxygen species and Ca^2+^ signaling, are known to involve ThDP-dependent enzymes^22^,^70^,^71^. Remarkably, thiamin and derivatives are also known to participate in biological responses to oxidative stress both as reactive oxygen species (ROS) scavengers^72^,^73^ and regulators of gene expression^74^,^75^. In view of the independent confirmation of the majority of the STRING-identified thiamin-dependent clusters, the abundance of 14-3-3 proteins and the presence of their different subunits and interacting proteins in both the thiamin and thiazolium proteomes (Supplementary Table S1, Fig. S2) favor a direct and biologically significant interaction of 14-3-3 proteins with thiamin.

The result of our manual search of the data pointed to multiple links between thiamin and the proteins identified in both the thiamin and thiazolium proteomes (Supplementary Table S1). The automatic proteome analysis with different bioinformatics tools supported the confidence that co-occurrence of the identified proteins upon affinity chromatography is not random. Identification of the protein clusters in the eluates from the thiamin or DMHT sorbents (Supplementary Fig. S2) suggests the thiamin dependence of the corresponding pathways. The dependence may be realized through concerted conformational changes within supramolecular protein structures upon the thiamin or derivative binding to some of the components present in such structures. In particular, multiple components of the redox, ubiquitinylation and signaling microcompartments including peroxiredoxins, DJ-1, amyloid-beta precursor and 14-3-3 proteins are revealed in the thiamin and thiazolium proteomes (Table 3, Supplementary Fig. S2).

### Thiamin-dependent regulation of the malate-aspartate shuttle

**Figure 9:**
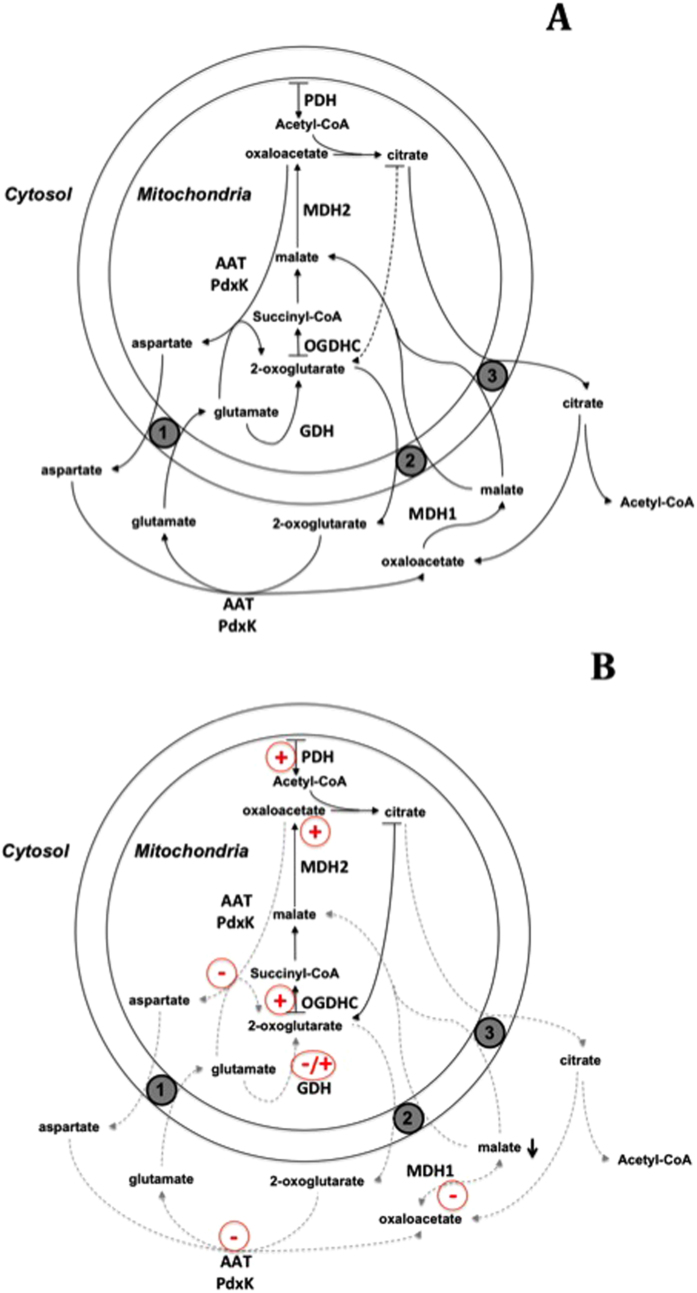
Thiamin-dependent regulation of the tricarboxylic acid (TCA) cycle and associated exchange of intermediates between cytoplasm and mitochondria: the malate-aspartate shuttle and the citrate-dependent transfer of acetyl-CoA. (**A**)—Distribution of fluxes at a lower thiamin level, when it mainly acts as the coenzyme of ThDP-dependent enzymes (K_d_ ∼ 10^−6^–10^−7^ M). (**B**)—Metabolic switch to increased flux through the TCA cycle in the presence of an excess of thiamin and derivatives exerting a non-coenzyme action (K_d_ ∼ 10^−5^ M). Stimulation and inhibition of the enzymes by thiamin and/or derivatives are shown by plus and minus annotations near the corresponding reactions. Effects on MDH 1 and MDH2 refer to low saturation of the enzymes with oxaloacetate. Effects on GDH depend on saturation with substrates and the present thiamin derivative, as shown in Fig. 6 and described in the text.

Figure 9 integrates our data into physiological context. The thiamin-dependent regulation of cytosolic MDH1 is directed to inhibit the cytosolic malate production (Fig. 6C). This inhibition is especially pronounced at low oxaloacetate concentration (Fig. 6D). Concomitant thiamin inhibition of PdxK (Figs 4B,7 and 8, Table 5) may further decrease oxaloacetate levels due to the decrease of pyridoxal phosphate level for the aspartate aminotransferase reaction. Indeed, a direct transfer of synthesized pyridoxal phosphate between the kinase and the aminotransferase has been hypothesized^76^, which is independently supported by our data. That is, a lower abundance of aminotransferase in the thiamin and/or thiazolium proteomes, compared to PdxK (Supplementary Table S1) suggests that the interaction of aspartate aminotransferase with the two sorbents was mediated through the heterologous complex of the enzyme with pyridoxal kinase, detected in both proteomes (Tables 2 and 3). The decrease in cytosolic malate concentration due to the thiamin-dependent inhibition of cytosolic MDH1 and PdxK would inhibit the malate transport into mitochondria. In other words, high thiamin/ThDP levels in cytoplasm may decrease the efficiency of malate-aspartate shuttling of reducing equivalents between mitochondria and cytoplasm (Fig. 9). Worth noting, regulation of GDH by thiamin compounds depends on the enzyme saturation with 2-oxoglutarate (Fig. 6E,F). This finding links the GDH regulation by thiamin to the malate-aspartate shuttle, as the shuttle involves 2-oxoglutarate as an intermediate (Fig. 9). Complexity of allosteric regulation of GDH and existence of multiple binding modes for the enzyme nucleotide substrates and regulators^64^ certainly requires future detailed studies of the possible interplay between the thiamin compounds and the known regulators of the GDH function.

It must be noted that, due to unfavorable equilibrium, the physiological directions of the reactions catalyzed by both GDH and mitochondrial MDH2, are opposite to those used in the standard assays of these enzymes *in vitro*. It is particularly important in this regard that the irreversible processes shifting the equilibrium of the physiological reactions of MDH2 (malate oxidation) and GDH (2-oxoglutarate production), involve ThDP-dependent pyruvate and 2-oxoglutarate dehydrogenases (Fig. 9). Thus, the enzymes revealed to be thiamin-dependent in this work are immediate metabolic neighbors of the known ThDP-dependent enzymes of central metabolism. The non-coenzyme action of thiamin on MDH1, MDH2, PdxK and GDH is thus coupled to the catalytic action of thiamin as a coenzyme. As a result, the concerted regulation of flux distributions through the TCA cycle and associated pathways may be achieved, as shown in Fig. 9 and discussed below.

### Implications of the protein targets of the non-coenzyme action of thiamin in acetylation processes, including the acetylation-dependent metabolic regulation and acetylcholine synthesis

One of the common features revealed with the highest statistical significance in both thiamin and thiazolium proteomes is the link of these proteomes to acetylation (Table 4). In the recent years, this post-translational modification of proteins has acquired increasing attention in view of its general regulatory importance for cellular metabolism^77^ and fate^78^. In contrast, the enrichment in the thiamin and thiazolium proteomes of the term *nucleotide binding* is characterized by tenth of orders of magnitude lower P-values (Table 4). This indicates that the thiamin and DMHT baits did not fish out the proteins which possess the binding sites for nicotinamide and adenine nucleotides, even if partial overlapping of such sites in the thiamin-dependent enzymes is possible (Fig. 5). Physiological significance of the highest enrichment of the annotation term *acetylation* in the thiamin and thiazolium proteomes is further supported by the known metabolic role of the acetylation of MDH1 and MDH2^79^,^80^, which we revealed to be thiamin targets as well. Remarkably, the intracellular compartmentalization of the cellular acetylating agent, acetyl-CoA, should be affected by the discussed above thiamin regulation of the malate fluxes. Produced by the ThDP-dependent pyruvate dehydrogenase reaction in mitochondria (Fig. 9), acetyl-CoA is transferred to cytoplasm in the form of citrate, which involves the citrate exchange for cytosolic malate (Fig. 9). Besides, citrate synthesis in mitochondria from acetyl-CoA requires oxaloacetate. According to the results of this work, also the latter is generated in a thiamin-dependent manner (Fig. 9). As shown in Fig. 9, the thiamin-induced decrease in formation of cytosolic malate should inhibit the exchange of mitochondrial citrate for cytosolic malate (Fig. 9B), increasing the citrate flux through the mitochondrial TCA cycle instead. Insofar, the allosteric action of thiamin may underlie the otherwise paradoxical effect of thiamin as an inhibitor of the acetylcholine synthesis in synaptosomes, which was mimicked by DMHT^81^. The existence of this regulatory mechanism of thiamin action may also explain facilitation of synaptic transmission, observed upon the thiamin co-release with acetylcholine^9^,^10^,^11^. That is, the neurotransmission-associated changes in intracellular thiamin concentration may be employed to couple metabolism and neurotransmission through oscillatory switching of fluxes between the TCA cycle and the associated pathway of acetyl-CoA transport from mitochondria to cytoplasm (Fig. 9). Regulatory acetylation of proteins may add to the kinetic effects of the coupled action of thiamin compounds as coenzyme and allosteric regulators, presented in Fig. 9.

### Multiple interactions between metabolic and signaling systems in the thiamin and thiazolium proteomes

Metabolic nodes involving the branched-point metabolites pyruvate, 2-oxoglutarate and citrate are important for overall metabolic regulation. In particular, recent studies revealed strong networking of the 2-oxoglutarate dehydrogenase-catalyzed degradation of 2-oxoglutarate with amino acids metabolism and protein homeostasis^22^. Another study showed that 2-oxoglutarate regulates the activity of TOR (Target Of Rapamycin) through the inhibitory binding to ATP synthase^82^. An interplay between thiamin deficiency and TOR activity has also been recently shown^83^. The thiamin-dependent coupling between metabolic network and signal transduction has been further supported by our manual search (Tables 2 and 3) and bioinformatics analysis (Supplementary Fig. S2, Fig. 1, Table 4) of the thiamin and thiazolium proteomes. The interactome analysis pinpointed possible links between the metabolic and regulatory pathways through 14-3-3 proteins, Ca^2+^ signaling and redox homeostasis (Supplementary Fig. S2), supported by published experimental data (Tables 2 and 3). For instance, protein-protein interaction between 14-3-3 proteins and glutamine synthetase^84^, proteins essential for neurotransmission and relatively abundant in our proteomes (Supplementary Table S1), could underlie concomitant oxidation of 14-3-3 proteins and glutamine synthetase upon oxidative stress induced by β-amyloid^85^. On the other hand, multiple links between physiological action of thiamin and cellular redox status are known. The ThDP-dependent 2-oxo acid dehydrogenase complexes were shown to be physiologically relevant sources of not only NADH, but also ROS production^86^,^87^,^88^. The ThDP-dependent transketolase is known to be essential for cellular NAD(P)H reducing potential. Moreover, experimentally confirmed functional interaction of the 2-oxo acid dehydrogenase complexes with thioredoxin^89^ was suggested to involve mitochondrial superoxide dismutase 2 (SOD2) and peroxiredoxins^70^, proteins which are present in the thiamin and/or thiazolium proteomes (Supplementary Table S1). The identification of DJ-1 and amyloid beta precursor protein AP4 in the thiazolium proteome (Supplementary Table S1) may be linked to the antioxidant cluster of the thiamin and thiazolium proteomes, supporting the thiamin significance in neurodegenerative diseases beyond the NAD(P)H production by ThDP-dependent enzymes. The analysis of the thiamin and thiazolium proteomes pursued in the present work provides new level of understanding of the molecular mechanisms of thiamin non-coenzyme function, extending the list of potential pharmacologically relevant pathways in the vitamin B1-employing therapies.

## Additional Information

**How to cite this article**: Mkrtchyan, G. *et al.* Molecular mechanisms of the non-coenzyme action of thiamin in brain: biochemical, structural and pathway analysis. *Sci. Rep.* **5**, 12583; doi: 10.1038/srep12583 (2015).

## Supplementary Material

Supplementary Information

## Figures and Tables

**Table 1:** Affinity purification of the thiamin phosphate hydrolyzing activity (ThDPase) from the acetone-delipidated synaptosomal proteins of rat brain.

Bait	Brain FW, g	Mass of affinity sorbent, g	Protein applied, mg	Protein yield and specific activity (nmol phosphate/min per mg of protein) eluted			
				1 M NaCl		2 М urea	
				mg/%	TDPase	mg/%	TDPase
Thiamin	25	0.8	110	1.5/1.4	200	1.1/1.3	3.2
DMHT	25	0.7	90	0.3/0.4	92	1.1/1.2	27

**Table 2:** Identification of the ThDP-dependent enzymes, their protein interactors and other proteins known to interact with thiamin and/or derivatives, in the eluates from thiamin (T)- and DMHT-modified sorbents.

Protein ID	Protein name	Proteome		Interaction partner	Source
		T	DHMT		
ODO1_RAT	2-oxoglutarate dehydrogenase, mitochondrial	−	+	ThDP	
ODPA_RAT	Pyruvate dehydrogenase E1 component subunit alpha, somatic form, mitochondrial	−	+	ThDP	
ODPB_RAT	Pyruvate dehydrogenase E1 component subunit beta, mitochondrial	−	+	ThDP	
TKT_RAT	Transketolase	+	+	ThDP	
ALBU_RAT	Serum albumin	+	+	Thiamin	90
ATPB_RAT	ATP synthase subunit beta, mitochondrial	−	+	ThDP, ThTP	34
HBA_RAT	Hemoglobin subunit alpha-1/2	+	+	Thiamin	72
HBB1_RAT	Hemoglobin subunit beta-1	+	+	Thiamin	72
HBB2_RAT	Hemoglobin subunit beta-2	+	+	Thiamin	72
DHE3_RAT	Glutamate dehydrogenase 1, mitochondrial	−	+	OGDHC	91
DLDH_RAT	Dihydrolipoyl dehydrogenase, mitochondrial	−	+	OGDHC, PDHC	
SUCA_RAT	Succinyl-CoA ligase [GDP-forming] subunit alpha, mitochondrial	+	−	OGDHC	92
CISY_RAT	Citrate synthase	−	+	PDHC	92
HSP72_RAT	Heat shock-related 70 kDa protein 2	−	+	OGDHC	93
HSP7C_RAT	Heat shock cognate 71 kDa protein	+	+	OGDHC	93
CH60_RAT	60 kDa heat shock protein, mitochondrial	−	+	OGDHC	93

The protein partners were included in the table, based on confirmed physical interactions according to published data. OGDHC, 2-oxoglutarate dehydrogenase complex; PDHC, pyruvate dehydrogenase complex.

**Table 3:** Physical and functional interactions between the proteins of the thiamin and thiazolium proteomes of rat brain synaptosomes.

Protein name	Interaction partner	Proteome		References	Type of interaction
		T	Tz		
Malate dehydrogenase (mitochondrial)	Glutamate dehydrogenase	−	+	94	Protein-protein interactions
	Citrate synthase	−	+	95	Protein-protein interactions
	Aspartate aminotransferase (mitochondrial)	+	+	96	Protein-protein interactions
Glutamate dehydrogenase	Malate dehydrogenase (mitochondrial)	−	+	94	Protein-protein interactions
	Aspartate aminotransferase (mitochondrial)	−	+	94	Protein-protein interactions
Pyridoxal kinase	Aspartate aminotransferase	+	+	97	Protein-protein interactions
14-3-3	Pyridoxal kinase	+	+	98,99	Protein-protein interactions
	Profilin	−	+	100	Protein-protein interactions
	Actin	+	+	100	Protein-protein interactions
	Tubulin	−	+	100	Protein-protein interactions
	PP1-alfa	−	+	98	Protein-protein interactions
	Casein kinase II	+	−	98	Protein-protein interactions
	Peroxiredoxin 2	−	+	98	Protein-protein interactions
	Peroxiredoxin 5	−	+	98	Protein-protein interactions
	Peroxiredoxin 6	−	+	98	Protein-protein interactions
	HSP70	+	+	98	Protein-protein interactions
	Glutamine synthase	+	−	84,101	Protein-protein interactions Functional interaction: concomitant oxidation of 14-3-3 and glutamine synthase in Amyloid beta neurotoxicity
Calmodulin	V-type ATPase, subunit A	−	+	102	Protein-protein interactions
	BASP1	−	+	103	Protein-protein interactions
	MARCKS	−	+	104	Protein-protein interactions
	Synapsin	−	+	105	Protein-protein interactions
V-type ATPase (subunits A, B, D, E1)	Aldolase A, C	+	−	106	Protein-protein interactions
BASP1	MARCKS	−	+	107	Protein-protein interactions
	Actin	−	+	108	Protein-protein interactions
MARCKS	HSP70	−	+	109	Protein-protein interactions
	Actin	−	+	108	Protein-protein interactions
Synapsin	Rab3A	−	+	110	Functional interaction; Rab3A inhibited synapsin I binding to F-actin, as well as synapsin-induced actin bundling and vesicle clustering
	Spectrin	−	+	111	Protein-protein interactions
	F-actin	−	+	112	Protein-protein interaction in a phosphorylation-dependent manner
Peroxiredoxin 6	Glutation-S-transferase Pi (GST Pi)	−	+	113	Functional interaction: oxidation of the catalytic cysteine in Prdx6 is required for its interaction with GST Pi
	Amyloid β A4 protein	−	+	114	Functional interaction: Prdx 6 protects PC12 cells from Aβ25-35-induced neurotoxicity
Amyloid β A4 protein	Serum Albumin	−	+	115	Protein-protein interactions
	Protein phosphatase 2A (PP2A)	−	+	116	Protein-protein interactions (with amyloid β)
Glutamate receptor 2 (GRIA2)	Tubulin	−	+	117	Protein-protein interactions
	WW domain-binding protein 2 (WWP2)	+	−	118	Functional interaction: ADAR2 protein levels are regulated by WWP2 and this may have downstream effects on GRIA2
	Casein kinase II	+	−	119	Functional interaction: casein kinase II phosphorylates GRIA2
Ubiquitin-dependent proteins	Ubiquitin carboxyl-terminal hydrolase 1	−	+	120	Proteins and targets of ubiquitin system
	Actin, cytoplasmic	+	+		
	Tubulin, alpha chain	+	+		
	Heat shock cognate 71 kDa protein	+	+		
	Dihydropyrimidinase-related protein 2	+	+		
	14-3-3 protein zeta/delta	+	+		
	Guanine nucleotide-binding protein G(o) subunit alpha	−	+		
	Synapsin-2	−	+		
	Amyloid β A4 protein	−	+		
Endophilin	Synaptojanin	−	+	121	Protein-protein interactions
	PP1-alpha	−	+	122	Protein-protein interactions
Aldose reductase	Hydroxyacylglutathione hydrolase mitochondrial (glyoxalase II)	+	−	123	Aldose reductase, glyoxalase I,and glyoxalase II are involved in the metabolism of methylglyoxal
	Tubulin	−	+	124	Protein-protein interactions

HSP70, Heat shock 70 kDa protein; BASP1, Brain acid soluble protein 1; MARCKS, Myristoylated alanine-rich C-kinase substrate.

**Table 4:** Functional annotation of the thiamin and thiazolium proteomes of rat brain synaptosomes in comparison to published partial brain proteomes of DJ-1-binding proteins^32^ and frontal cortex^33^.

Proteome											
Thiamin (54)			Thiazolium (144)			Dj-1 (312)			Frontal cortex (395)		
**Annotation term**	**n**	**P-value**	**Annotation term**	**n**	**P-value**	**Annotation term**	**n**	**P-value**	**Annotation term**	**n**	**P-value**
1. Acetylation	33	9*10^−17^	1. Acetylation	87	4*10^−41^	1. Acetylation	146	10^−89^	1. Phospho-protein	250	2*10^−31^
2. Oxygen carrier	4	5*10^−7^	2. Phosphoprotein	97	2*10^−20^	2. Ribonucleo-protein	41	3*10^−34^	2. Coiled coil	92	9*10^−16^
4. Phospho-protein	33	2*10^−6^	3. Cytoplasm	61	4*10^−14^	3. Phospho-protein	143	1*10^−27^	4. Nucleus	132	8*10^−9^
8. Nucleotide-binding	12	10^−4^	7. Nucleotide-binding	34	2*10^−6^	11. Nucleotide-binding	45	9*10^−9^	14. Nucleotide-binding	64	5*10^−6^

DJ-1 protein was detected in the thiazolium proteome, whereas proteome of frontal cortex represents independently purified fraction of brain proteins. The total number of proteins in the proteomes: thiamin—57, thiazolium—150, Dj-1 (parkin7)—755, frontal cortex—412. The total numbers of genes accepted by DAVID from the identified proteomes are indicated in the table in parenthesis. SP_PIR_KEYWORDS category was used to characterize the proteomes. The first two annotation terms which are most significantly enriched, are given for each proteome. The third term with significant P-value is chosen according to the protein number n indicating how many proteins of the proteomes relate to this term. Nucleotide-binding annotation term is included to show a relatively low significance of enrichment of the thiamin and thiazolium proteomes with the nucleotide binding proteins. The number preceding the term name corresponds to the position of its P-value in the DAVID-generated output.

**Table 5:** Parameters and mechanisms of inhibition of PdxK by thiamin and ThDP.

Inhibitor	Variable substrate	Fixed substrate	Type of inhibition	K_i_(mM)
Thiamin	Pyridoxal	MgATP	Competitive	2.2
	MgATP	Pyridoxal	Non-competitive	6.6
ThDP	Pyridoxal	MgATP	Non-competitive	10.4
	MgATP	Pyridoxal	Competitive	3.3
