# Supplementary material for: Molecular mechanisms of the non-coenzyme action of thiamin in brain: biochemical, structural and pathway analysis
Source: Sci Rep. 2015 Jul 27;5:12583. doi: 10.1038/srep12583 (PMC4515825; doi:10.1038/srep12583)
Supplement: Supplementary Information [file srep12583-s1.docx]

**Molecular mechanisms of the non-coenzyme action of thiamin in brain: biochemical, structural and pathway analysis**

Mkrtchyan Garik, Aleshin Vasily, Parkhomenko Yulia, Kaehne Thilo, di Salvo Martino Luigi, Parroni Alessia, Contestabile Roberto, Vovk Andrey, Bettendorff Lucien, Bunik Victoria

**Supplementary information**


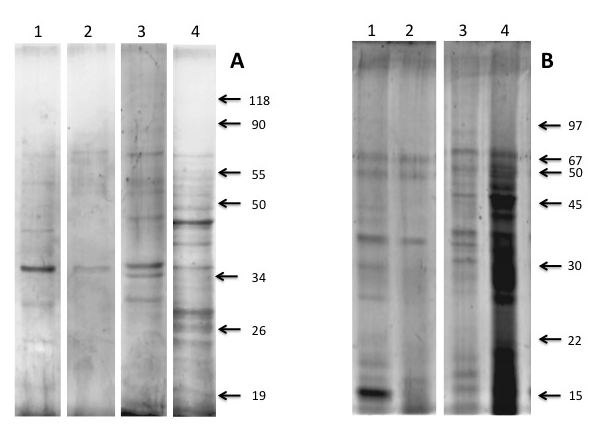


**Supplementary Fig. S1**. SDS-electrophoresis of proteins eluted from the affine sorbents modified by thiamin (lanes 1,2) and DMHT (lanes 3,4). The proteins were eluted by 10 mM Tris-HCl, pH 7.4, with 1M NaCl (lanes 1,3) and 2 M urea (lanes 2,4). 1 µg of the eluted protein (A, 7% gel) or equal volume (15 µL) of each fraction (B, 10% gel) was applied to lanes 1-4. Position of markers on the gels is given by the arrows with molecular masses in kDa.

Unprocessed original scans of SDS gels for Supplementary Fig. S1A and B are provided below as gels A’ and B’ with the lanes 1 – 4 indicated, correspondently.

**Supplementary Fig. S2**. STRING visualization of the thiamine (A) and thiazolium (B) interactomes, defined by the database-extracted interactions between the identified proteins of the proteomes. Color code for the interactions is given on the figure. Encircled protein clusters correspond to metabolism (blue), signaling through 14–3–3 proteins (red), Ca^2+^-dependent regulation (yellow), redox state (green).

**Supplementary Fig. S3.** Thiamine and derivatives binding patterns in the 3D structures of MDH from archaea (**A**) and bacteria (**B**). Close view of the thiamin diphosphokinase/ThiB pattern (pink) found in different sequence parts of the archaeal and bacterial MDHs is presented together with the patterns found in eukaryotic MDHs (yellow and orange) to show localization of all these patterns close to the active site. The pattern color code and other details are as in Fig. 3. **A** - Dimer of MDH from *Haloarcula marismortui,* R207S, R292S mutant with NAD^+^ bound (PDB ID: 2X0R). **B** – Dimer of MDH from *Aquaspirillium arcticum* with NAD^+^ and oxaloacetate (colored in green) bound (PDB ID: 1B8U).

**Supplementary Table S1**

Accession codes of proteins are given according to Mnemonic identifiers of UniProtKB

**A – Thiamin-binding proteome**

| **Accession** | **Protein** | **MW [kDa]** | **pI** | **Scores** | **#Peptides** | **SC [%]** | **repeats** |
| --- | --- | --- | --- | --- | --- | --- | --- |
| **MDHM_RAT** | **Malate dehydrogenase, mitochondrial OS=Rattus norvegicus GN=Mdh2 PE=1 SV=2** | **35,7** | **9,8** | **1130,4** | **16** | **56,5** | **9** |
| **PDXK_RAT** | **Pyridoxal kinase OS=Rattus norvegicus GN=Pdxk PE=1 SV=1** | **34,9** | **6,4** | **933,6** | **11** | **50** | **3** |
| PDIA3_RAT | Protein disulfide-isomerase A3 OS=Rattus norvegicus GN=Pdia3 PE=1 SV=2 | 56,6 | 5,8 | 717,6 | 13 | 26,5 | 2 |
| HSP7C_RAT | Heat shock cognate 71 kDa protein OS=Rattus norvegicus GN=Hspa8 PE=1 SV=1 | 70,8 | 5,2 | 684 | 10 | 20,9 | 3 |
| SYYC_RAT | Tyrosyl-tRNA synthetase, cytoplasmic OS=Rattus norvegicus GN=Yars PE=2 SV=3 | 59,1 | 6,6 | 607,9 | 11 | 20,8 | 0 |
| CYC_RAT | Cytochrome c, somatic OS=Rattus norvegicus GN=Cycs PE=1 SV=2 | 11,6 | 10,2 | 588,4 | 8 | 59 | 0 |
| ALBU_RAT | Serum albumin OS=Rattus norvegicus GN=Alb PE=1 SV=2 | 68,7 | 6,1 | 586,6 | 12 | 20,9 | 6 |
| HBB2_RAT | Hemoglobin subunit beta-2 OS=Rattus norvegicus PE=1 SV=2 | 16 | 9,7 | 508 | 9 | 63,3 | 1 |
| PPIA_RAT | Peptidyl-prolyl cis-trans isomerase A OS=Rattus norvegicus GN=Ppia PE=1 SV=2 | 17,9 | 9,4 | 491,2 | 10 | 33,5 | 1 |
| SODM_RAT | Superoxide dismutase [Mn], mitochondrial OS=Rattus norvegicus GN=Sod2 PE=1 SV=2 | 24,7 | 9,65 | 476,17 | 7 | 38,7 | 1 |
| HBB1_RAT | Hemoglobin subunit beta-1 OS=Rattus norvegicus GN=Hbb PE=1 SV=3 | 16 | 9 | 451,6 | 2 | 51,7 | 3 |
| 1433Z_RAT | 14-3-3 protein zeta/delta OS=Rattus norvegicus GN=Ywhaz PE=1 SV=1 | 27,8 | 4,57 | 444,62 | 6 | 27,3 | 0 |
| 1433E_RAT | 14-3-3 protein epsilon OS=Rattus norvegicus GN=Ywhae PE=1 SV=1 | 29,2 | 4,48 | 347,7 | 6 | 24,7 | 1 |
| K1C10_RAT | Keratin, type I cytoskeletal 10 OS=Rattus norvegicus GN=Krt10 PE=2 SV=1 | 56,5 | 5 | 341,7 | 5 | 8,9 | 4 |
| GDIR1_RAT | Rho GDP-dissociation inhibitor 1 OS=Rattus norvegicus GN=Arhgdia PE=1 SV=1 | 23,4 | 5 | 329 | 5 | 35,3 | 1 |
| HBA_RAT | Hemoglobin subunit alpha-1/2 OS=Rattus norvegicus GN=Hba1 PE=1 SV=3 | 15,3 | 9 | 313,8 | 4 | 41,5 | 2 |
| RADI_MOUSE | Radixin OS=Mus musculus GN=Rdx PE=1 SV=2 | 68,6 | 5,8 | 307,2 | 9 | 11,7 | 0 |
| EZRI_RAT | Ezrin OS=Rattus norvegicus GN=Ezr PE=1 SV=3 | 69,3 | 5,8 | 299,8 | 3 | 2,7 | 0 |
| GRP78_RAT | 78 kDa glucose-regulated protein OS=Rattus norvegicus GN=Hspa5 PE=1 SV=1 | 72,3 | 4,92 | 286,51 | 3 | 8,7 | 0 |
| DHPR_RAT | Dihydropteridine reductase OS=Rattus norvegicus GN=Qdpr PE=1 SV=1 | 25,5 | 8,99 | 278,93 | 3 | 29,9 | 0 |
| PSTS_ECOLI | Phosphate-binding protein pstS OS=Escherichia coli (strain K12) GN=pstS PE=1 SV=1 | 37 | 9 | 261,2 | 4 | 11,8 | 0 |
| SPTA2_RAT | Spectrin alpha chain, brain OS=Rattus norvegicus GN=Sptan1 PE=1 SV=2 | 284,5 | 5,08 | 245,1 | 2 | 4,3 | 0 |
| TRIM2_MOUSE | Tripartite motif-containing protein 2 OS=Mus musculus GN=Trim2 PE=1 SV=1 | 81,4 | 6,5 | 242,2 | 5 | 6,6 | 0 |
| GSTP1_RAT | Glutathione S-transferase P OS=Rattus norvegicus GN=Gstp1 PE=1 SV=2 | 23,4 | 7,69 | 215,85 | 2 | 15,2 | 0 |
| 1433G_RAT | 14-3-3 protein gamma OS=Rattus norvegicus GN=Ywhag PE=1 SV=2 | 28,3 | 4,65 | 213,61 | 4 | 15 | 0 |
| GSTM4_RAT | Glutathione S-transferase Yb-3 OS=Rattus norvegicus GN=Gstm3 PE=1 SV=2 | 25,7 | 7,63 | 210,87 | 3 | 20,2 | 0 |
| SUCA_RAT | Succinyl-CoA ligase [GDP-forming] subunit alpha, mitochondrial OS=Rattus norvegicus GN=Suclg1 PE=2 SV=2 | 36,1 | 10,29 | 203,48 | 5 | 19,4 | 0 |
| GLO2_RAT | Hydroxyacylglutathione hydrolase, mitochondrial OS=Rattus norvegicus GN=Hagh PE=1 SV=2 | 34,1 | 8,78 | 192,19 | 3 | 13,3 | 0 |
| OMPA_SERMA | Outer membrane protein A OS=Serratia marcescens GN=ompA PE=3 SV=1 | 38,4 | 8,9 | 179,8 | 4 | 13,1 | 0 |
| KACA_RAT | Ig kappa chain C region, A allele OS=Rattus norvegicus PE=1 SV=1 | 11,7 | 4,85 | 173,85 | 2 | 32,1 | 0 |
| TKT_RAT | Transketolase OS=Rattus norvegicus GN=Tkt PE=1 SV=1 | 67,6 | 7,9 | 172,4 | 5 | 6,4 | 1 |
| CAH2_RAT | Carbonic anhydrase 2 OS=Rattus norvegicus GN=Ca2 PE=1 SV=2 | 29,1 | 7,1 | 168,5 | 4 | 14,2 | 1 |
| ECI1_RAT | Enoyl-CoA delta isomerase 1, mitochondrial OS=Rattus norvegicus GN=Eci1 PE=1 SV=1 | 32,2 | 10,06 | 166,91 | 3 | 13,5 | 0 |
| NSF_RAT | Vesicle-fusing ATPase OS=Rattus norvegicus GN=Nsf PE=1 SV=1 | 82,6 | 6,6 | 163,6 | 3 | 4,4 | 2 |
| ACTG_RAT | Actin, cytoplasmic 2 OS=Rattus norvegicus GN=Actg1 PE=1 SV=1 | 41,8 | 5,2 | 161,3 | 2 | 11,7 | 0 |
| ALDOC_RAT | Fructose-bisphosphate aldolase C OS=Rattus norvegicus GN=Aldoc PE=1 SV=3 | 39,3 | 6,82 | 158,6 | 2 | 8,5 | 0 |
| AATC_RAT | Aspartate aminotransferase, cytoplasmic OS=Rattus norvegicus GN=Got1 PE=1 SV=3 | 46,4 | 6,9 | 149,8 | 2 | 5,3 | 0 |
| MANF_RAT | Mesencephalic astrocyte-derived neurotrophic factor OS=Rattus norvegicus GN=Manf PE=1 SV=1 | 20,4 | 9,5 | 137,3 | 3 | 16,8 | 1 |
| CF203_MOUSE | Uncharacterized protein C6orf203 homolog OS=Mus musculus PE=1 SV=1 | 27,8 | 9,9 | 133,4 | 4 | 10 | 0 |
| RP3A_RAT | Rabphilin-3A OS=Rattus norvegicus GN=Rph3a PE=1 SV=1 | 75,8 | 9,4 | 128 | 3 | 5,4 | 0 |
| GLNA_RAT | Glutamine synthetase OS=Rattus norvegicus GN=Glul PE=1 SV=3 | 42,2 | 6,7 | 117,8 | 3 | 7,2 | 1 |
| ALDOA_RAT | Fructose-bisphosphate aldolase A OS=Rattus norvegicus GN=Aldoa PE=1 SV=2 | 39,3 | 9,2 | 115,2 | 2 | 5,8 | 1 |
| ECH1_RAT | Delta(3,5)-Delta(2,4)-dienoyl-CoA isomerase, mitochondrial OS=Rattus norvegicus GN=Ech1 PE=1 SV=2 | 36,1 | 9,14 | 109,73 | 1 | 8,9 | 0 |
| DPYL2_MOUSE | Dihydropyrimidinase-related protein 2 OS=Mus musculus GN=Dpysl2 PE=1 SV=2 | 62,2 | 5,9 | 103,3 | 2 | 3 | 0 |
| CSK21_RAT | Casein kinase II subunit alpha OS=Rattus norvegicus GN=Csnk2a1 PE=1 SV=2 | 45 | 7,9 | 100,3 | 2 | 6,4 | 1 |
| LDHA_RAT | L-lactate dehydrogenase A chain OS=Rattus norvegicus GN=Ldha PE=1 SV=1 | 36,4 | 9,3 | 91,5 | 2 | 5,7 | 0 |
| SEPT7_RAT | Septin-7 OS=Rattus norvegicus GN=Sept7 PE=1 SV=1 | 50,5 | 9,5 | 91,2 | 2 | 5,5 | 0 |
| ALDR_RAT | Aldose reductase OS=Rattus norvegicus GN=Akr1b1 PE=1 SV=3 | 35,8 | 6,3 | 89,5 | 2 | 6,3 | 0 |
| GRIA2_RAT | Glutamate receptor 2 OS=Rattus norvegicus GN=Gria2 PE=1 SV=2 | 98,6 | 7,66 | 87,46 | 2 | 6,3 | 0 |
| THY1_RAT | Thy-1 membrane glycoprotein OS=Rattus norvegicus GN=Thy1 PE=1 SV=1 | 18,2 | 10,4 | 87,2 | 2 | 10,6 | 1 |
| IGG2B_RAT | Ig gamma-2B chain C region OS=Rattus norvegicus GN=Igh-1a PE=1 SV=1 | 36,5 | 8,9 | 85 | 2 | 5,4 | 0 |
| ACTB_RAT | Actin, cytoplasmic 1 OS=Rattus norvegicus GN=Actb PE=1 SV=1 | 41,7 | 5,18 | 81,49 | 1 | 5,6 | 0 |
| ACV1C_RAT | Activin receptor type-1C OS=Rattus norvegicus GN=Acvr1c PE=1 SV=1 | 54,8 | 8,96 | 81,45 | 2 | 5,1 | 0 |
| PP1A_MOUSE | Serine/threonine-protein phosphatase PP1-alpha catalytic subunit OS=Mus musculus GN=Ppp1ca PE=1 SV=1 | 37,5 | 5,9 | 78,7 | 2 | 6,1 | 0 |
| MGT5A_RAT | Alpha-1,6-mannosylglycoprotein 6-beta-N-acetylglucosaminyltransferase A OS=Rattus norvegicus GN=Mgat5 PE=1 SV=1 | 84,5 | 9,39 | 73,31 | 2 | 5,3 | 0 |
| OTC_RAT | Ornithine carbamoyltransferase, mitochondrial OS=Rattus norvegicus GN=Otc PE=1 SV=1 | 39,9 | 9,56 | 49,72 | 1 | 3,4 | 0 |

**B – Thiazolium-binding proteome**

| **Accession** | **Protein** | **MW [kDa]** | **pI** | **Scores** | **#Peptides** | **SC [%]** | **repeats** |
| --- | --- | --- | --- | --- | --- | --- | --- |
| ALBU_RAT | Serum albumin OS=Rattus norvegicus GN=Alb PE=1 SV=2 | 68,7 | 6,08 | 1193,73 | 18 | 32,1 | 17 |
| **MDHM_RAT** | **Malate dehydrogenase, mitochondrial OS=Rattus norvegicus GN=Mdh2 PE=1 SV=2** | **35,7** | **9,83** | **1134,82** | **16** | **61,2** | **16** |
| ACTB_RAT | Actin, cytoplasmic 1 OS=Rattus norvegicus GN=Actb PE=1 SV=1 | 41,7 | 5,18 | 1024,05 | 14 | 51,2 | 23 |
| TBA1A_RAT | Tubulin alpha-1A chain OS=Rattus norvegicus GN=Tuba1a PE=1 SV=1 | 50,1 | 4,81 | 976,43 | 13 | 51,9 | 8 |
| TBA1B_RAT | Tubulin alpha-1B chain OS=Rattus norvegicus GN=Tuba1b PE=1 SV=1 | 50,1 | 4,81 | 958,66 | 13 | 51,9 | 4 |
| ACTG_RAT | Actin, cytoplasmic 2 OS=Rattus norvegicus GN=Actg1 PE=1 SV=1 | 41,8 | 5,2 | 953,52 | 11 | 48 | 11 |
| 1433E_RAT | 14-3-3 protein epsilon OS=Rattus norvegicus GN=Ywhae PE=1 SV=1 | 29,2 | 4,48 | 934,15 | 12 | 52,9 | 15 |
| **PDXK_RAT** | **Pyridoxal kinase OS=Rattus norvegicus GN=Pdxk PE=1 SV=1** | **34,9** | **6,35** | **907,78** | **12** | **55,8** | **12** |
| VATB2_RAT | V-type proton ATPase subunit B, brain isoform OS=Rattus norvegicus GN=Atp6v1b2 PE=1 SV=1 | 56,5 | 5,48 | 865,05 | 11 | 34,8 | 12 |
| 1433Z_RAT | 14-3-3 protein zeta/delta OS=Rattus norvegicus GN=Ywhaz PE=1 SV=1 | 27,8 | 4,57 | 863,88 | 11 | 57,6 | 13 |
| **DHE3_RAT** | **Glutamate dehydrogenase 1, mitochondrial OS=Rattus norvegicus GN=Glud1 PE=1 SV=2** | **61,4** | **8,84** | **815,73** | **11** | **32,8** | **17** |
| GLNA_RAT | Glutamine synthetase OS=Rattus norvegicus GN=Glul PE=1 SV=3 | 42,2 | 6,73 | 808,79 | 9 | 40,2 | 10 |
| ALDOA_RAT | Fructose-bisphosphate aldolase A OS=Rattus norvegicus GN=Aldoa PE=1 SV=2 | 39,3 | 9,2 | 760,55 | 11 | 37,6 | 11 |
| VATA_MOUSE | V-type proton ATPase catalytic subunit A OS=Mus musculus GN=Atp6v1a PE=1 SV=2 | 68,3 | 5,3 | 727,1 | 12 | 20,1 | 5 |
| HS90A_RAT | Heat shock protein HSP 90-alpha OS=Rattus norvegicus GN=Hsp90aa1 PE=1 SV=3 | 84,8 | 4,78 | 727,08 | 11 | 22 | 13 |
| DLDH_RAT | Dihydrolipoyl dehydrogenase, mitochondrial OS=Rattus norvegicus GN=Dld PE=1 SV=1 | 54 | 8,98 | 724,94 | 8 | 35,8 | 4 |
| TBA4A_RAT | Tubulin alpha-4A chain OS=Rattus norvegicus GN=Tuba4a PE=2 SV=1 | 49,9 | 4,79 | 705,34 | 10 | 37,1 | 1 |
| GDIA_RAT | Rab GDP dissociation inhibitor alpha OS=Rattus norvegicus GN=Gdi1 PE=1 SV=1 | 50,5 | 4,86 | 668,15 | 9 | 30 | 5 |
| CALB1_RAT | Calbindin OS=Rattus norvegicus GN=Calb1 PE=1 SV=2 | 30 | 4,56 | 650,77 | 9 | 44,1 | 2 |
| ATPB_RAT | ATP synthase subunit beta, mitochondrial OS=Rattus norvegicus GN=Atp5b PE=1 SV=2 | 56,3 | 5,1 | 640,1 | 10 | 18,9 | 9 |
| GSTM1_RAT | Glutathione S-transferase Mu 1 OS=Rattus norvegicus GN=Gstm1 PE=1 SV=2 | 25,9 | 9,01 | 578,77 | 10 | 56 | 3 |
| ACTC_RAT | Actin, alpha cardiac muscle 1 OS=Rattus norvegicus GN=Actc1 PE=1 SV=1 | 42 | 5,11 | 565,41 | 7 | 33,2 | 1 |
| PPIA_RAT | Peptidyl-prolyl cis-trans isomerase A OS=Rattus norvegicus GN=Ppia PE=1 SV=2 | 17,9 | 9,42 | 558,94 | 8 | 61 | 3 |
| TBB2A_RAT | Tubulin beta-2A chain OS=Rattus norvegicus GN=Tubb2a PE=1 SV=1 | 49,9 | 4,64 | 538,9 | 6 | 26,1 | 2 |
| ALDOC_RAT | Fructose-bisphosphate aldolase C OS=Rattus norvegicus GN=Aldoc PE=1 SV=3 | 39,3 | 6,82 | 531,54 | 6 | 32,2 | 6 |
| HBB1_RAT | Hemoglobin subunit beta-1 OS=Rattus norvegicus GN=Hbb PE=1 SV=3 | 16 | 9,05 | 527,1 | 8 | 64,6 | 5 |
| ACTS_RAT | Actin, alpha skeletal muscle OS=Rattus norvegicus GN=Acta1 PE=2 SV=1 | 42 | 5,11 | 518,62 | 6 | 26,3 | 3 |
| HS90B_RAT | Heat shock protein HSP 90-beta OS=Rattus norvegicus GN=Hsp90ab1 PE=1 SV=4 | 83,2 | 4,82 | 518,57 | 9 | 17,4 | 9 |
| ACTA_RAT | Actin, aortic smooth muscle OS=Rattus norvegicus GN=Acta2 PE=1 SV=1 | 42 | 5,12 | 506,71 | 8 | 23,3 | 2 |
| C1QBP_RAT | Complement component 1 Q subcomponent-binding protein, mitochondrial OS=Rattus norvegicus GN=C1qbp PE=1 SV=2 | 31 | 4,63 | 490,28 | 5 | 22,2 | 2 |
| TBB2C_RAT | Tubulin beta-2C chain OS=Rattus norvegicus GN=Tubb2c PE=1 SV=1 | 49,8 | 4,65 | 478,74 | 7 | 17,3 | 1 |
| HSP7C_RAT | Heat shock cognate 71 kDa protein OS=Rattus norvegicus GN=Hspa8 PE=1 SV=1 | 70,8 | 5,24 | 467,91 | 6 | 13,3 | 4 |
| ACT17_DICDI | Actin-17 OS=Dictyostelium discoideum GN=act17 PE=3 SV=1 | 41,5 | 5,2 | 466,2 | 2 | 20,9 | 0 |
| GSTM4_RAT | Glutathione S-transferase Yb-3 OS=Rattus norvegicus GN=Gstm3 PE=1 SV=2 | 25,7 | 7,63 | 463,54 | 6 | 35,3 | 1 |
| 1433G_RAT | 14-3-3 protein gamma OS=Rattus norvegicus GN=Ywhag PE=1 SV=2 | 28,3 | 4,65 | 456,13 | 6 | 28,7 | 7 |
| KCRB_RAT | Creatine kinase B-type OS=Rattus norvegicus GN=Ckb PE=1 SV=2 | 42,7 | 5,31 | 453,09 | 6 | 22,8 | 16 |
| TBA1C_RAT | Tubulin alpha-1C chain OS=Rattus norvegicus GN=Tuba1c PE=1 SV=1 | 49,9 | 4,83 | 451,68 | 6 | 18,5 | 3 |
| AATM_RAT | Aspartate aminotransferase, mitochondrial OS=Rattus norvegicus GN=Got2 PE=1 SV=2 | 47,3 | 9,81 | 444,05 | 6 | 17 | 1 |
| PRDX1_RAT | Peroxiredoxin-1 OS=Rattus norvegicus GN=Prdx1 PE=1 SV=1 | 22,1 | 9,2 | 442,5 | 10 | 44,2 | 3 |
| CALB2_RAT | Calretinin OS=Rattus norvegicus GN=Calb2 PE=1 SV=1 | 31,4 | 4,8 | 440,28 | 6 | 35,1 | 1 |
| SSDH_RAT | Succinate-semialdehyde dehydrogenase, mitochondrial OS=Rattus norvegicus GN=Aldh5a1 PE=1 SV=2 | 56,1 | 9,2 | 424,8 | 6 | 17 | 11 |
| 1433F_RAT | 14-3-3 protein eta OS=Rattus norvegicus GN=Ywhah PE=1 SV=2 | 28,2 | 4,66 | 407,61 | 5 | 23,2 | 6 |
| TBA3_RAT | Tubulin alpha-3 chain OS=Rattus norvegicus GN=Tuba3a PE=2 SV=1 | 49,9 | 4,84 | 404,51 | 6 | 20 | 1 |
| CISY_RAT | Citrate synthase, mitochondrial OS=Rattus norvegicus GN=Cs PE=1 SV=1 | 51,8 | 9,22 | 404,46 | 5 | 17,8 | 1 |
| CYC_RAT | Cytochrome c, somatic OS=Rattus norvegicus GN=Cycs PE=1 SV=2 | 11,6 | 10,19 | 404,09 | 6 | 55,2 | 2 |
| COF1_RAT | Cofilin-1 OS=Rattus norvegicus GN=Cfl1 PE=1 SV=3 | 18,5 | 9,13 | 399,45 | 6 | 42,2 | 0 |
| SODM_RAT | Superoxide dismutase [Mn], mitochondrial OS=Rattus norvegicus GN=Sod2 PE=1 SV=2 | 24,7 | 9,65 | 395,67 | 5 | 32,9 | 0 |
| HBA_RAT | Hemoglobin subunit alpha-1/2 OS=Rattus norvegicus GN=Hba1 PE=1 SV=3 | 15,3 | 9,05 | 392,77 | 5 | 52,1 | 1 |
| DHPR_RAT | Dihydropteridine reductase OS=Rattus norvegicus GN=Qdpr PE=1 SV=1 | 25,5 | 9 | 384,2 | 5 | 48,1 | 2 |
| PP2BA_MOUSE | Serine/threonine-protein phosphatase 2B catalytic subunit alpha isoform OS=Mus musculus GN=Ppp3ca PE=1 SV=1 | 58,6 | 5,5 | 375,7 | 6 | 9 | 3 |
| ARP2_RAT | Actin-related protein 2 OS=Rattus norvegicus GN=Actr2 PE=1 SV=1 | 44,7 | 6,3 | 368,4 | 4 | 14,7 | 2 |
| HBB2_RAT | Hemoglobin subunit beta-2 OS=Rattus norvegicus PE=1 SV=2 | 16 | 9,66 | 366 | 5 | 42,2 | 1 |
| LDHA_RAT | L-lactate dehydrogenase A chain OS=Rattus norvegicus GN=Ldha PE=1 SV=1 | 36,4 | 9,3 | 364,6 | 6 | 21,1 | 2 |
| GBB1_RAT | Guanine nucleotide-binding protein G(I)/G(S)/G(T) subunit beta-1 OS=Rattus norvegicus GN=Gnb1 PE=1 SV=4 | 37,4 | 5,56 | 347,98 | 5 | 27,6 | 0 |
| 1433B_RAT | 14-3-3 protein beta/alpha OS=Rattus norvegicus GN=Ywhab PE=1 SV=3 | 28 | 4,66 | 341,14 | 5 | 26,3 | 7 |
| SYNJ1_RAT | Synaptojanin-1 OS=Rattus norvegicus GN=Synj1 PE=1 SV=3 | 172,8 | 6,45 | 338,66 | 4 | 7,6 | 3 |
| PROF2_RAT | Profilin-2 OS=Rattus norvegicus GN=Pfn2 PE=1 SV=3 | 15 | 7,47 | 337,53 | 5 | 54,3 | 0 |
| TBB2B_RAT | Tubulin beta-2B chain OS=Rattus norvegicus GN=Tubb2b PE=1 SV=1 | 49,9 | 4,64 | 333,73 | 6 | 19,3 | 0 |
| ARPC2_RAT | Actin-related protein 2/3 complex subunit 2 OS=Rattus norvegicus GN=Arpc2 PE=1 SV=1 | 34,4 | 7 | 325,6 | 8 | 22,3 | 2 |
| DPYL2_RAT | Dihydropyrimidinase-related protein 2 OS=Rattus norvegicus GN=Dpysl2 PE=1 SV=1 | 62,2 | 5,94 | 325,2 | 4 | 10,5 | 6 |
| FAAA_RAT | Fumarylacetoacetase OS=Rattus norvegicus GN=Fah PE=1 SV=1 | 45,9 | 6,76 | 321,53 | 5 | 15 | 1 |
| GFAP_RAT | Glial fibrillary acidic protein OS=Rattus norvegicus GN=Gfap PE=1 SV=2 | 49,9 | 5,23 | 316,89 | 5 | 13 | 2 |
| PPIB_RAT | Peptidyl-prolyl cis-trans isomerase B OS=Rattus norvegicus GN=Ppib PE=2 SV=3 | 23,8 | 10,1 | 312,58 | 5 | 26,4 | 0 |
| GLO2_RAT | Hydroxyacylglutathione hydrolase, mitochondrial OS=Rattus norvegicus GN=Hagh PE=1 SV=2 | 34,1 | 8,78 | 311,91 | 4 | 20,1 | 2 |
| PARK7_RAT | Protein DJ-1 OS=Rattus norvegicus GN=Park7 PE=1 SV=1 | 20 | 6,4 | 311,4 | 7 | 29,6 | 2 |
| RCN2_RAT | Reticulocalbin-2 OS=Rattus norvegicus GN=Rcn2 PE=1 SV=2 | 37,4 | 4,12 | 297,56 | 4 | 24,4 | 0 |
| ALDR_RAT | Aldose reductase OS=Rattus norvegicus GN=Akr1b1 PE=1 SV=3 | 35,8 | 6,3 | 294,1 | 7 | 17,7 | 2 |
| CH60_RAT | 60 kDa heat shock protein, mitochondrial OS=Rattus norvegicus GN=Hspd1 PE=1 SV=1 | 60,9 | 5,84 | 289,72 | 3 | 15,9 | 1 |
| BASP1_RAT | Brain acid soluble protein 1 OS=Rattus norvegicus GN=Basp1 PE=1 SV=2 | 21,8 | 4,33 | 287,36 | 4 | 55,9 | 3 |
| CSK2B_RAT | Casein kinase II subunit beta OS=Rattus norvegicus GN=Csnk2b PE=1 SV=1 | 24,9 | 5,24 | 286,03 | 5 | 34 | 0 |
| 1433T_RAT | 14-3-3 protein theta OS=Rattus norvegicus GN=Ywhaq PE=1 SV=1 | 27,8 | 4,54 | 283,8 | 4 | 27,8 | 0 |
| ARF3_RAT | ADP-ribosylation factor 3 OS=Rattus norvegicus GN=Arf3 PE=1 SV=2 | 20,6 | 7,7 | 272,35 | 4 | 29,8 | 1 |
| HPLN1_RAT | Hyaluronan and proteoglycan link protein 1 OS=Rattus norvegicus GN=Hapln1 PE=1 SV=2 | 40,2 | 9,1 | 267,7 | 6 | 23,4 | 0 |
| K6PF_RAT | 6-phosphofructokinase, muscle type OS=Rattus norvegicus GN=Pfkm PE=2 SV=3 | 85,5 | 9,18 | 251,83 | 5 | 12,8 | 2 |
| PRDX2_RAT | Peroxiredoxin-2 OS=Rattus norvegicus GN=Prdx2 PE=1 SV=3 | 21,8 | 5,24 | 249,64 | 3 | 23,2 | 1 |
| A4_RAT | Amyloid beta A4 protein OS=Rattus norvegicus GN=App PE=1 SV=2 | 86,6 | 4,58 | 240,43 | 3 | 6,4 | 5 |
| PP2BA_RAT | Serine/threonine-protein phosphatase 2B catalytic subunit alpha isoform OS=Rattus norvegicus GN=Ppp3ca PE=1 SV=1 | 58,6 | 5,51 | 236,44 | 3 | 7,3 | 2 |
| GSTM5_RAT | Glutathione S-transferase Mu 5 OS=Rattus norvegicus GN=Gstm5 PE=1 SV=3 | 26,6 | 6,41 | 234,19 | 3 | 27,6 | 0 |
| CALM_RAT | Calmodulin OS=Rattus norvegicus GN=Calm1 PE=1 SV=2 | 16,8 | 3,93 | 232,68 | 2 | 26,2 | 0 |
| MARCS_RAT | Myristoylated alanine-rich C-kinase substrate OS=Rattus norvegicus GN=Marcks PE=1 SV=2 | 29,8 | 4,2 | 232,5 | 4 | 25,6 | 3 |
| TBA8_RAT | Tubulin alpha-8 chain OS=Rattus norvegicus GN=Tuba8 PE=2 SV=1 | 50 | 4,84 | 228,96 | 3 | 14,9 | 0 |
| LIS1_PIG | Platelet-activating factor acetylhydrolase IB subunit alpha OS=Sus scrofa GN=PAFAH1B1 PE=2 SV=3 | 46,6 | 7,2 | 226,5 | 6 | 16,6 | 0 |
| ACADL_RAT | Long-chain specific acyl-CoA dehydrogenase, mitochondrial OS=Rattus norvegicus GN=Acadl PE=1 SV=1 | 47,8 | 8,7 | 217,9 | 4 | 10,7 | 5 |
| UCHL1_RAT | Ubiquitin carboxyl-terminal hydrolase isozyme L1 OS=Rattus norvegicus GN=Uchl1 PE=1 SV=2 | 24,8 | 5,01 | 214,99 | 4 | 29,1 | 1 |
| GABT_RAT | 4-aminobutyrate aminotransferase, mitochondrial OS=Rattus norvegicus GN=Abat PE=1 SV=3 | 56,4 | 9,16 | 214,9 | 3 | 13,6 | 1 |
| SPON1_RAT | Spondin-1 OS=Rattus norvegicus GN=Spon1 PE=1 SV=1 | 90,7 | 5,78 | 214 | 3 | 9,8 | 0 |
| CSK21_RAT | Casein kinase II subunit alpha OS=Rattus norvegicus GN=Csnk2a1 PE=1 SV=2 | 45 | 7,87 | 204,73 | 4 | 10 | 3 |
| PPM1A_RAT | Protein phosphatase 1A OS=Rattus norvegicus GN=Ppm1a PE=1 SV=1 | 42,4 | 5,1 | 203,8 | 5 | 14,4 | 1 |
| GNAO_RAT | Guanine nucleotide-binding protein G(o) subunit alpha OS=Rattus norvegicus GN=Gnao1 PE=1 SV=2 | 40 | 5,2 | 200,6 | 4 | 18,1 | 2 |
| FKB1A_RAT | Peptidyl-prolyl cis-trans isomerase FKBP1A OS=Rattus norvegicus GN=Fkbp1a PE=2 SV=3 | 11,9 | 9,12 | 199,48 | 2 | 25 | 0 |
| CYTC_RAT | Cystatin-C OS=Rattus norvegicus GN=Cst3 PE=1 SV=2 | 15,4 | 10,19 | 196,12 | 2 | 20 | 3 |
| LEG1_RAT | Galectin-1 OS=Rattus norvegicus GN=Lgals1 PE=1 SV=2 | 14,8 | 4,96 | 195,88 | 3 | 23,7 | 0 |
| PGK1_RAT | Phosphoglycerate kinase 1 OS=Rattus norvegicus GN=Pgk1 PE=1 SV=2 | 44,5 | 9,01 | 191,47 | 2 | 10,1 | 1 |
| AL9A1_RAT | 4-trimethylaminobutyraldehyde dehydrogenase OS=Rattus norvegicus GN=Aldh9a1 PE=1 SV=1 | 53,6 | 6,7 | 187 | 4 | 8,3 | 0 |
| SEPT7_RAT | Septin-7 OS=Rattus norvegicus GN=Sept7 PE=1 SV=1 | 50,5 | 9,5 | 177 | 3 | 7,6 | 2 |
| BDH2_RAT | 3-hydroxybutyrate dehydrogenase type 2 OS=Rattus norvegicus GN=Bdh2 PE=3 SV=2 | 26,6 | 7,61 | 173,27 | 2 | 14,3 | 2 |
| PP2AB_RAT | Serine/threonine-protein phosphatase 2A catalytic subunit beta isoform OS=Rattus norvegicus GN=Ppp2cb PE=1 SV=1 | 35,6 | 5,1 | 171,54 | 3 | 16,5 | 0 |
| ACTN4_RAT | Alpha-actinin-4 OS=Rattus norvegicus GN=Actn4 PE=1 SV=2 | 104,8 | 5,16 | 168,3 | 2 | 5,3 | 1 |
| RAB3A_MOUSE | Ras-related protein Rab-3A OS=Mus musculus GN=Rab3a PE=1 SV=1 | 25 | 4,7 | 167,8 | 3 | 15 | 0 |
| CAH2_RAT | Carbonic anhydrase 2 OS=Rattus norvegicus GN=Ca2 PE=1 SV=2 | 29,1 | 7,1 | 167 | 4 | 14,2 | 0 |
| ANXA5_RAT | Annexin A5 OS=Rattus norvegicus GN=Anxa5 PE=1 SV=3 | 35,7 | 4,8 | 160,5 | 3 | 9,1 | 0 |
| GSTP1_RAT | Glutathione S-transferase P OS=Rattus norvegicus GN=Gstp1 PE=1 SV=2 | 23,4 | 7,69 | 160,19 | 2 | 12,9 | 3 |
| ODPB_RAT | Pyruvate dehydrogenase E1 component subunit beta, mitochondrial OS=Rattus norvegicus GN=Pdhb PE=1 SV=2 | 39 | 6,2 | 157,5 | 3 | 8,9 | 0 |
| TBB5_RAT | Tubulin beta-5 chain OS=Rattus norvegicus GN=Tubb5 PE=1 SV=1 | 49,6 | 4,64 | 155,05 | 3 | 7,2 | 1 |
| PP2AA_MOUSE | Serine/threonine-protein phosphatase 2A catalytic subunit alpha isoform OS=Mus musculus GN=Ppp2ca PE=1 SV=1 | 35,6 | 5,2 | 152,9 | 3 | 8,7 | 0 |
| TPIS_RAT | Triosephosphate isomerase OS=Rattus norvegicus GN=Tpi1 PE=1 SV=2 | 26,8 | 7,7 | 152,1 | 3 | 14,1 | 1 |
| ARL3_RAT | ADP-ribosylation factor-like protein 3 OS=Rattus norvegicus GN=Arl3 PE=1 SV=2 | 20,4 | 7,6 | 149,3 | 3 | 18,1 | 0 |
| PHYIP_RAT | Phytanoyl-CoA hydroxylase-interacting protein OS=Rattus norvegicus GN=Phyhip PE=2 SV=1 | 37,5 | 6,59 | 144,18 | 2 | 8,2 | 1 |
| HSP72_RAT | Heat shock-related 70 kDa protein 2 OS=Rattus norvegicus GN=Hspa2 PE=2 SV=2 | 69,6 | 5,38 | 141,68 | 2 | 3,8 | 2 |
| NDKB_RAT | Nucleoside diphosphate kinase B OS=Rattus norvegicus GN=Nme2 PE=1 SV=1 | 17,3 | 7,78 | 137,03 | 3 | 19,7 | 0 |
| WBP2_RAT | WW domain-binding protein 2 OS=Rattus norvegicus GN=Wbp2 PE=1 SV=1 | 28,1 | 5,9 | 136,5 | 3 | 11,5 | 2 |
| TKT_RAT | Transketolase OS=Rattus norvegicus GN=Tkt PE=1 SV=1 | 67,6 | 7,9 | 132,5 | 4 | 5,5 | 0 |
| TRY1_RAT | Anionic trypsin-1 OS=Rattus norvegicus GN=Prss1 PE=1 SV=1 | 25,9 | 4,56 | 129,34 | 1 | 8,1 | 0 |
| ERP44_MOUSE | Endoplasmic reticulum resident protein 44 OS=Mus musculus GN=Erp44 PE=1 SV=1 | 46,8 | 5 | 122,8 | 3 | 6,9 | 0 |
| CYBP_RAT | Calcyclin-binding protein OS=Rattus norvegicus GN=Cacybp PE=1 SV=1 | 26,5 | 8,72 | 122,64 | 2 | 10,9 | 0 |
| ODPA_RAT | Pyruvate dehydrogenase E1 component subunit alpha, somatic form, mitochondrial OS=Rattus norvegicus GN=Pdha1 PE=1 SV=2 | 43,2 | 9,37 | 119,38 | 2 | 6,2 | 0 |
| ODO1_RAT | 2-oxoglutarate dehydrogenase, mitochondrial OS=Rattus norvegicus GN=Ogdh PE=1 SV=1 | 116,2 | 6,31 | 114,26 | 2 | 4,3 | 0 |
| TPM3_RAT | Tropomyosin alpha-3 chain OS=Rattus norvegicus GN=Tpm3 PE=1 SV=2 | 29 | 4,6 | 111,6 | 2 | 8,5 | 0 |
| TERA_RAT | Transitional endoplasmic reticulum ATPase OS=Rattus norvegicus GN=Vcp PE=1 SV=3 | 89,3 | 5 | 106,01 | 2 | 6,2 | 0 |
| GBB4_RAT | Guanine nucleotide-binding protein subunit beta-4 OS=Rattus norvegicus GN=Gnb4 PE=2 SV=4 | 37,3 | 5,7 | 105,8 | 2 | 6,2 | 0 |
| NDKA_RAT | Nucleoside diphosphate kinase A OS=Rattus norvegicus GN=Nme1 PE=1 SV=1 | 17,2 | 5,93 | 104,91 | 2 | 20,4 | 0 |
| ARP3_RAT | Actin-related protein 3 OS=Rattus norvegicus GN=Actr3 PE=1 SV=1 | 47,3 | 5,5 | 102,7 | 2 | 10,8 | 0 |
| NUDC_RAT | Nuclear migration protein nudC OS=Rattus norvegicus GN=Nudc PE=1 SV=1 | 38,4 | 5,15 | 102,17 | 2 | 6,9 | 0 |
| DPYL5_RAT | Dihydropyrimidinase-related protein 5 OS=Rattus norvegicus GN=Dpysl5 PE=1 SV=1 | 61,5 | 6,7 | 101,9 | 1 | 5,3 | 1 |
| VATE1_RAT | V-type proton ATPase subunit E 1 OS=Rattus norvegicus GN=Atp6v1e1 PE=1 SV=1 | 26,1 | 9,11 | 101,67 | 1 | 6,2 | 0 |
| NMT1_RAT | Glycylpeptide N-tetradecanoyltransferase 1 OS=Rattus norvegicus GN=Nmt1 PE=2 SV=1 | 56,8 | 8,83 | 100,26 | 1 | 2,8 | 1 |
| PRDX5_RAT | Peroxiredoxin-5, mitochondrial OS=Rattus norvegicus GN=Prdx5 PE=1 SV=1 | 22,2 | 10,08 | 99,9 | 1 | 15,5 | 1 |
| KPYM_HUMAN | Pyruvate kinase isozymes M1/M2 OS=Homo sapiens GN=PKM2 PE=1 SV=4 | 57,9 | 9 | 97,9 | 2 | 4,1 | 0 |
| PGCB_RAT | Brevican core protein OS=Rattus norvegicus GN=Bcan PE=1 SV=2 | 96 | 4,7 | 97,1 | 2 | 2 | 0 |
| CSN5_MOUSE | COP9 signalosome complex subunit 5 OS=Mus musculus GN=Cops5 PE=1 SV=3 | 37,5 | 6,1 | 95,1 | 2 | 5,1 | 0 |
| GBB2_RAT | Guanine nucleotide-binding protein G(I)/G(S)/G(T) subunit beta-2 OS=Rattus norvegicus GN=Gnb2 PE=1 SV=4 | 37,3 | 5,56 | 94 | 1 | 11,2 | 0 |
| CHM4B_MOUSE | Charged multivesicular body protein 4b OS=Mus musculus GN=Chmp4b PE=2 SV=2 | 24,9 | 4,6 | 93,4 | 2 | 11,2 | 1 |
| PP2BB_RAT | Serine/threonine-protein phosphatase 2B catalytic subunit beta isoform OS=Rattus norvegicus GN=Ppp3cb PE=2 SV=1 | 59,1 | 5,5 | 92,3 | 2 | 4,2 | 0 |
| GBB3_RAT | Guanine nucleotide-binding protein G(I)/G(S)/G(T) subunit beta-3 OS=Rattus norvegicus GN=Gnb3 PE=1 SV=1 | 37,2 | 5,44 | 91,17 | 1 | 5,3 | 0 |
| RLA1_RAT | 60S acidic ribosomal protein P1 OS=Rattus norvegicus GN=Rplp1 PE=3 SV=1 | 11,5 | 4,07 | 90,1 | 1 | 19,3 | 1 |
| PRDX6_RAT | Peroxiredoxin-6 OS=Rattus norvegicus GN=Prdx6 PE=1 SV=3 | 24,8 | 5,6 | 88,6 | 2 | 8 | 0 |
| THIO_RAT | Thioredoxin OS=Rattus norvegicus GN=Txn PE=1 SV=2 | 11,7 | 4,64 | 87,24 | 1 | 12,4 | 0 |
| SH3G2_RAT | Endophilin-A1 OS=Rattus norvegicus GN=Sh3gl2 PE=1 SV=2 | 39,9 | 5,1 | 86,3 | 2 | 5,4 | 0 |
| UBP1_RAT | Ubiquitin carboxyl-terminal hydrolase 1 OS=Rattus norvegicus GN=Usp1 PE=2 SV=1 | 87,3 | 5,2 | 85,3 | 1 | 7,1 | 0 |
| SAHH_RAT | Adenosylhomocysteinase OS=Rattus norvegicus GN=Ahcy PE=1 SV=3 | 47,5 | 6,1 | 84,2 | 2 | 4,9 | 0 |
| CRK_RAT | Adapter molecule crk OS=Rattus norvegicus GN=Crk PE=1 SV=1 | 33,8 | 5,28 | 83,32 | 1 | 7,9 | 2 |
| UBP5_HUMAN | Ubiquitin carboxyl-terminal hydrolase 5 OS=Homo sapiens GN=USP5 PE=1 SV=2 | 95,7 | 4,8 | 82,6 | 2 | 2,7 | 0 |
| MANF_RAT | Mesencephalic astrocyte-derived neurotrophic factor OS=Rattus norvegicus GN=Manf PE=1 SV=1 | 20,4 | 9,5 | 81,6 | 1 | 11,2 | 0 |
| SYN2_RAT | Synapsin-2 OS=Rattus norvegicus GN=Syn2 PE=1 SV=1 | 63,4 | 9,4 | 78,6 | 2 | 4,9 | 0 |
| RS4X_HUMAN | 40S ribosomal protein S4, X isoform OS=Homo sapiens GN=RPS4X PE=1 SV=2 | 29,6 | 10,8 | 77,8 | 2 | 6,8 | 0 |
| PYGB_RAT | Glycogen phosphorylase, brain form (Fragment) OS=Rattus norvegicus GN=Pygb PE=1 SV=3 | 96,1 | 6,2 | 68,8 | 2 | 2 | 0 |
| VATD_MOUSE | V-type proton ATPase subunit D OS=Mus musculus GN=Atp6v1d PE=1 SV=1 | 28,4 | 9,9 | 62,7 | 2 | 6,9 | 0 |
| THIM_RAT | 3-ketoacyl-CoA thiolase, mitochondrial OS=Rattus norvegicus GN=Acaa2 PE=1 SV=1 | 41,8 | 9,1 | 52,29 | 1 | 2 | 0 |
| DGLB_RAT | Sn1-specific diacylglycerol lipase beta OS=Rattus norvegicus GN=Daglb PE=1 SV=1 | 73,7 | 6,22 | 45,06 | 1 | 2,2 | 0 |
| OTC_RAT | Ornithine carbamoyltransferase, mitochondrial OS=Rattus norvegicus GN=Otc PE=1 SV=1 | 39,9 | 9,56 | 44,54 | 1 | 3,4 | 0 |
